# Topology- and wavelength-governed CO_2_ reduction photocatalysis in molecular catalyst-metal–organic framework assemblies†

**DOI:** 10.1039/d2sc03097g

**Published:** 2022-10-03

**Authors:** Philip M. Stanley, Karina Hemmer, Markus Hegelmann, Annika Schulz, Mihyun Park, Martin Elsner, Mirza Cokoja, Julien Warnan

**Affiliations:** Chair of Inorganic and Metal–Organic Chemistry, Department of Chemistry, TUM School of Natural Sciences, Catalysis Research Center (CRC), Technical University of Munich Garching Germany julien.warnan@tum.de; Chair of Analytical Chemistry and Water Chemistry, Department of Chemistry, TUM School of Natural Sciences, Technical University of Munich Garching Germany

## Abstract

Optimising catalyst materials for visible light-driven fuel production requires understanding complex and intertwined processes including light absorption and catalyst stability, as well as mass, charge, and energy transport. These phenomena can be uniquely combined (and ideally controlled) in porous host–guest systems. Towards this goal we designed model systems consisting of molecular complexes as catalysts and porphyrin metal–organic frameworks (MOFs) as light-harvesting and hosting porous matrices. Two MOF-rhenium molecule hybrids with identical building units but differing topologies (PCN-222 and PCN-224) were prepared including photosensitiser-catalyst dyad-like systems integrated *via* self-assembled molecular recognition. This allowed us to investigate the impact of MOF topology on solar fuel production, with PCN-222 assemblies yielding a 9-fold turnover number enhancement for solar CO_2_-to-CO reduction over PCN-224 hybrids as well as a 10-fold increase compared to the homogeneous catalyst-porphyrin dyad. Catalytic, spectroscopic and computational investigations identified larger pores and efficient exciton hopping as performance boosters, and further unveiled a MOF-specific, wavelength-dependent catalytic behaviour. Accordingly, CO_2_ reduction product selectivity is governed by selective activation of two independent, circumscribed or delocalised, energy/electron transfer channels from the porphyrin excited state to either formate-producing MOF nodes or the CO-producing molecular catalysts.

## Abstract 2

Two MOF molecular catalyst hybrids with differing topologies show mass and light transport governed photocatalysis. MOF-specific, irradiation wavelength-dependent product control is unlocked by switching between two energy/electron transfer channels.

## Graphical abstract

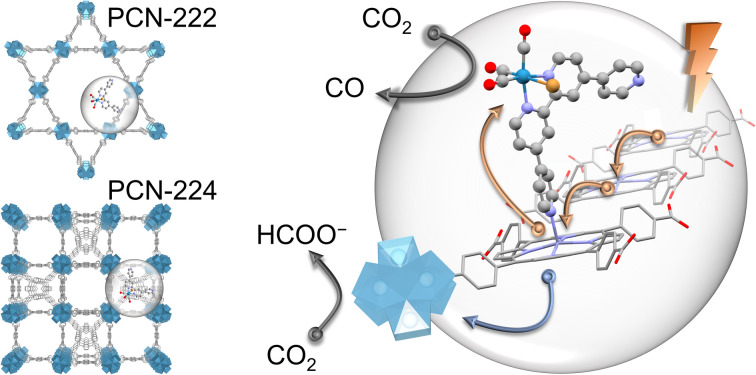

## Introduction

Against the backdrop of increasing global energy requirements, photocatalysis has the potential to reduce the necessary energy for feedstock production and to transform waste into valuable feedstocks.^1^ Solar fuel production, merging solar energy harvesting with subsequent chemical energy conversion, is a pertinent route towards such artificial photosynthesis.^2^ The latter motivates bio-inspired molecular catalysts, fine-tuned to maximise selectivity and atom efficiency while minimising activation energy barriers.^3,4^ However, these are often mired by limited stability and device integrability, as well as modest light-harvesting efficiency for photocatalytic systems operating under broad-spectrum irradiation.^1^ Accordingly, current efforts synergise material and catalyst co-design to enable energy-intense reactions, such as selective CO_2_ reduction, in light-absorbing hybrids.^3,4^

Here, metal–organic framework (MOF) materials show promise due to their highly controllable chemical and (photo)physical properties enabled by their modular assembly.^5^ This unlocks a breadth of differing porosities, topologies, pore sizes, guest inclusion opportunities, optoelectronic properties, and more, which intertwine and deeply condition reaction environments.^6–8^ Thus, combining molecular catalysts and MOFs to heterogeneous hybrids enables catalyst stabilisation and recyclability while also gaining control over its proximal and global environment.^7,9,10^ While previous reports on heterogeneous thermal MOF catalysis demonstrated that nanoreactors built by pore walls affect reaction activity, selectivity, and substrate diffusion to active sites,^8,11^ these findings are not generally transferable to systems with light-driven catalysis, cascade electroreductions or defined molecular complexes as the active species. Accordingly, challenges remain in the conceptual understanding of the intrinsic effects of a MOF pore environment on such molecular MOF hybrids towards rational design of effective materials for photocatalysis. Although a few studies have investigated pore size variation, topology-based substrate orientation, and active site distance optimisation, these parameters remain poorly understood and systematic studies on how MOF topology influences properties and solar fuel performances are needed.^12–14^

**Fig. 1:**
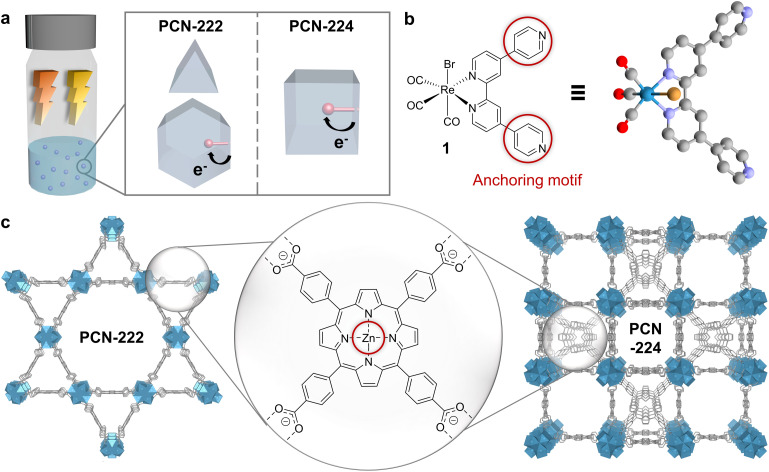
Components and structures of the assemblies. (a) Conceptual representation of the designed and synthesised materials to study the influence of MOF topology and irradiation wavelength on colloidal photocatalytic CO_2_ reduction with dyadic Re-based assemblies in PCN-222 and PCN-224. Blue spheres: MOFs. Red spheres: molecular catalyst. (b) Left: Chemical structure of ReBr(CO)_3_(qtpy) (1). Circles: Pyridine anchoring motif for dyad formation. Right: DFT-optimised structure of 1. Red: oxygen, grey: carbon, purple: N, gold: Br, cyan: Re. H atoms omitted for clarity. (c) Structures of the MOFs PCN-222 (left) and PCN-224 (right), showcasing their differing topologies but identical building blocks (porphyrin-based linkers and Zr_6_-oxo-based nodes). Red: Zn anchoring motif for dyadic formation *via* Zn–N_(pyridine)_ bonds. Rendered from CCDC 893545 and 1001133.

Herein, we designed two supramolecular MOF-catalyst hybrids constructed from identical building blocks but differing in topology (Fig. 1a). The assemblies consist of a *fac*-ReBr(CO)_3_(qtpy) (1, qtpy = 4,4′:2′,2′′:4,4′′′-quaterpyridine) CO_2_ reduction catalyst and Zn-metalated 5,10,15,20-tetrakis(4-carboxylphenyl)porphyrin (2-Zn) linker- and Zr_6_-oxo node-based MOFs, *i.e.*, PCN-222 and PCN-224 (Fig. 1b and c). These MOFs were chosen as model systems for their broad visible-light absorption,^15–18^ and their reported directional exciton migration and energy funnelling to acceptors.^19,20^ While 1 has not been applied in photocatalytic CO_2_ reduction to date, its derivatives are known for selective CO formation under homogeneous and heterogeneous conditions, with turnover numbers (TONs) ranging from ∼10 to 4500 in the presence of sacrificial electron donors (SEDs).^9,21–23^

The MOF hosts, PCN-222 and PCN-224, were synthesised with high crystallinity, Zn metalated and then post-synthetically linker-functionalised with 1. In contrast to prior time-consuming multistep synthesis of dyadic porphyrin photosensitiser (PS) and Re-based catalyst assemblies (*e.g.*, *via* alkyl chains, phenyl spacers, or amide group), here, interfacing was achieved in mild conditions through supramolecular Zn–N interactions.^24–28^ We took advantage of the penta-coordination preference of zinc in porphyrins and the strong affinity of azoarenes for Zn porphyrins to form a self-assembled dyadic system confined inside the porous network.^29^ Reminiscent of the Mg chlorophylls held by axial ligation of histidine on the natural light harvesting antennae protein scaffold, this approach enables the introduction of matching dyadic systems into two different MOF topologies towards monitoring the topology-catalysis relationships. Applying these Re-PCN colloids for photocatalytic CO_2_ reduction in acetonitrile (MeCN) with a SED showed a 9-fold TON_CO_ enhancement for PCN-222 over PCN-224 hybrids as well as a 10-fold increase compared to the homogeneous catalyst-porphyrin dyad. Characterisations by powder X-ray diffraction (PXRD), solution and solid-state UV-vis, and attenuated total reflectance infrared (ATR-IR) spectroscopy; inductively coupled plasma mass spectrometry (ICP-MS); N_2_ adsorption; density functional theory (DFT) calculations and difference envelope density (DED) analysis uncovered mechanistic insights controlling reaction rate (*via* topology-guided mass and exciton transport) and selectivity (*via* wavelength-enabled electron/energy channel switching).

## Results and discussion

### Synthesis and characterisation

The quaterpyridine ligand and *fac*-ReBr(CO)_3_(qtpy) (1) were synthesised following literature known procedures,^30,31^ while the linker 5,10,15,20-tetrakis(4-carboxyphenyl)porphyrin (2) was prepared by saponification of 5,10,15,20-tetrakis(4-methoxycarbonylphenyl)-porphyrin (TPPCOOMe), attained from pyrrole and methyl 4-formylbenzoate.^32^ The Zn-metalated analogue of 2 (2-Zn) was prepared by reacting TPPCOOMe with ZnCl_2_, followed by saponification (details in the ESI) for benchmarking purposes. PCN-222 (222) and PCN-224 (224) syntheses were adapted from literature and the as-synthesised MOFs were treated with HCl in *N*,*N*-dimethylformamide (DMF) at 120 °C for modulator removal.^14,33^ Zinc metalation of the free base porphyrin linkers was performed in DMF at 100 °C for 24 h resulting in PCN-222(Zn) (222-Zn) and PCN-224(Zn) (224-Zn) (details and characterisation available in ESI†).^34,35^

1 was integrated into 222-Zn and 224-Zn, respectively, by soaking the MOFs in a 0.1 mM stock solution of 1 in MeCN resulting in 1@PCN-222(Zn) (Re-222) and 1@PCN-224(Zn) (Re-224), respectively, and isolated as powders. Loading of 1 was monitored by UV-vis measurements of the supernatant which displayed a strong decline in the intensity of the absorption bands of 1 after 24 h (Fig. S1†). After washing the composites with MeCN, no absorption bands of the catalyst were visible in the UV-vis spectrum of the washing solution, excluding strong leaching of the catalyst and underscoring robust Zn–N interactions (Fig. S2†). Control experiments performed by soaking the Zn-free MOF 222 or 224 in a 0.1 mM solution of 1 showed no decrease in supernatant absorption, confirming the specific dyadic anchoring site of 1 at Zn centres (Fig. S3†).

**Fig. 2:**
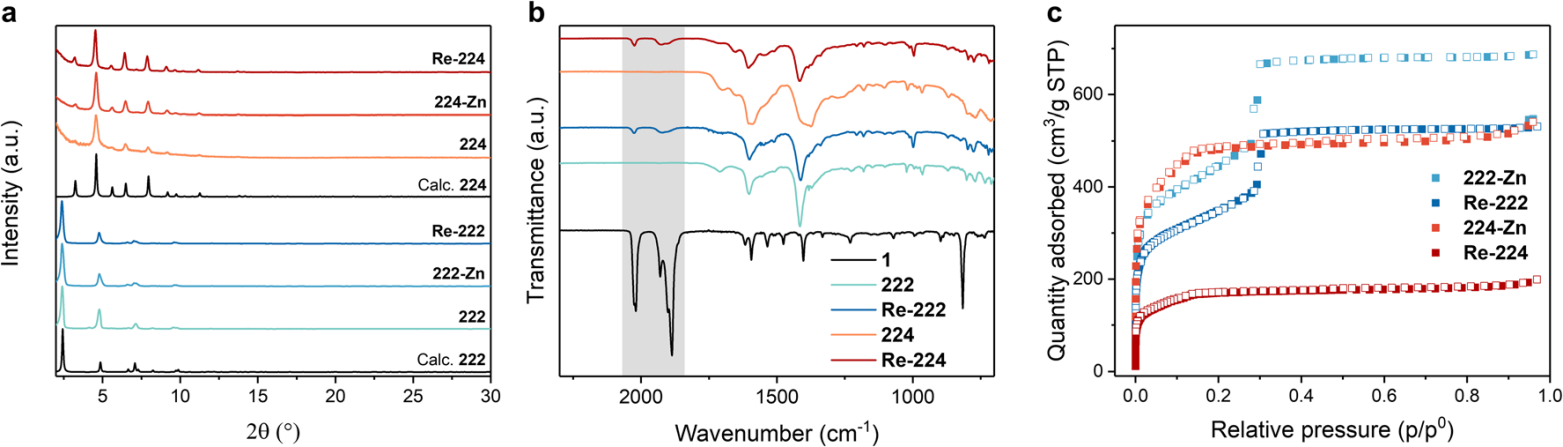
Material characterisation for pristine and loaded PCN-222 and PCN-224 assemblies. (a) Measured PXRD data. (b) ATR-IR spectra including the reference for pure catalyst 1 (black trace) and with the characteristic region for Re(CO)_3_ bands highlighted in grey. Full spectra in ESI.† (c) N_2_ sorption isotherms at 77 K.

The unaltered PXRD data of both MOFs upon Zn metalation and Re catalyst incorporation revealed preserved phase purity and crystallinity while being in accordance with the simulated patterns (Fig. 2a). The ATR-IR spectra of both Re-222 and Re-224 display symmetric and asymmetric CO vibrations at 1926 and 2025 cm^−1^ and 1927 and 2024 cm^−1^, respectively (Fig. 2b and S4†), characteristic of 1, and suggesting successful entrapment as well as unaffected molecular structure after immobilisation in the MOFs.^36^ Furthermore, the shift of Zn–N bands at ∼398 cm^−1^ after loading of 1 hints at a comprehensive coordination of the Zn porphyrins (Fig. S5†).^37^

Solid-state UV-vis spectra showed comprehensive absorption in the visible-light region for both metalated MOFs, highlighting the suitability of these materials for broad range visible light harvesting (Fig. S6 and S7†). While 222 and 224 display broadened absorption features in line with 2's characteristic Soret band at 420 nm and the Q-bands in the range of 510–650 nm (Fig. S8†), hallmark symmetry-restoring changes were observed upon Zn metalation potentially indicating quantitative Zn metalation. In contrast, incorporation of 1 does not affect the absorption spectra suggesting that visible light irradiation will mainly result in MOF host excitation (Fig. S1, S6 and S7†).

ICP-MS performed after MOF digestion of 222-Zn and 224-Zn revealed Zr : Zn ratios of 1.8 and 1.9, respectively, indicating that every porphyrin is likely metalated (as Zr : Zn_theoretical_ = 3 and 4, respectively, assuming reaction yield = 100%) and that additional Zn is physisorbed in the MOF pores (Table S1†). Together with the Zn-TCPP, these may act as additional anchoring sites for 1. Nevertheless, the Re content in Re-222 and Re-224 was found similar, ∼82 and ∼75 nmol_Re_ mg_MOF_^−1^, respectively, further confirming the successful incorporation of 1 in 222-Zn and 224-Zn (∼0.1 catalyst per linker in both cases, see Table S1†). Loading values are comparable to other molecule-MOF hybrids which often use carboxylic or phosphonic acids for node- or linker-anchoring.^9,12,19,38^ Accordingly, our approach provides a synthetically straightforward alternative anchoring design to traditional acid groups (increasing the design space and assembly flexibility) while still affording stable tethering and similar loadings.

Nitrogen adsorption measurements showed a reduction in the N_2_ quantity adsorbed for both 222-Zn and 224-Zn compared to the corresponding pristine MOFs. The Brunauer–Emmett–Teller (BET) surface area was reduced from ∼1990 to ∼1550 m^2^ g^−1^ for 222 and 222-Zn, and from ∼1980 to ∼1750 m^2^ g^−1^ for 224 and 224-Zn, respectively (Fig. S9 and S10†). Such a decrease upon metalation is common and has been observed for other PCN analogues.^8,34^ A further decrease of the N_2_ quantity adsorbed upon incorporating 1 is observable for both MOF topologies (Fig. 2c and S10†). The N_2_ quantity adsorbed in Re-224 is more dramatically reduced compared to Re-222, indicating a higher extent of pore blocking in the former case. This is further illustrated by the BET surface areas and calculated pore size distributions of ∼1219 m^2^ g^−1^ for Re-222, while a BET surface area of merely ∼618 m^2^ g^−1^ is obtained for Re-224. Scanning electron microscopy (SEM) images visualised rod-shaped ∼10 × 1 μm 222 crystals and cube-type ∼0.5 × 0.5 μm 224 crystals, in line with literature (Fig. S11 and S12†).^14^

Previous studies have reported on the measurements of conduction and valence band levels of PCN MOFs.^39^ Alternatively, a recent study on a PCN-molecular catalyst hybrid showed that complete isolation of the organic porphyrin linkers in a rigid structure with insulating metal nodes prevents an electronic coupling, concluding that excited energy levels of PCN MOFs can be regarded as those of the free linker.^19^ The oxidation potential of 2-Zn* (S_1_) was previously calculated at −1.24 V *vs.* saturated calomel electrode (*V*_SCE_).^19^ With *E*(1/1^−^) = −0.64 V_SCE_, photoinduced electron transfer from 2-Zn* to 1 in the PCN assemblies is thermodynamically possible, and was reported as the preferred first electron transfer step for Re-porphyrin dyads, as opposed to reductive quenching by the SED.^24,25,31^ In photocatalysis conditions, the corresponding oxidised PS at *E* = 0.88 V_SCE_ will react with 1,3-dimethyl-2-phenyl-2,3-dihydro-1*H*-benzo[*d*]imidazole (BIH) employed as a benign SED to regenerate the PS ground state with *E*(BIH^+^/BIH) = 0.33 V_SCE_ (Fig. S13†).^39,40^

In summary, assembly characterisation suggests similar catalyst loading and light-absorbing behaviour for both topologies, however, differing in permanent porosity and, accordingly, likely differing in substrate-catalyst accessibility.

### Photocatalysis under broadband irradiation

Photocatalysis experiments were conducted under heterogeneous colloidal conditions, namely suspended MOF assembly (1.5 mg) in wet organic media (4 mL MeCN and 0.12 mL deionised H_2_O) and BIH (225 mg, 1 mmol) as the SED. The resulting suspension was saturated by bubbling CO_2_. Irradiation was performed with a Xenon Light Source (300 W, *λ* = 430–740 nm, ∼10 mW cm^−2^). CO and H_2_ evolution were analysed by gas chromatography of the reaction headspace, while formate formation was investigated by ^1^H NMR of the reaction solution.

First, we examined the activity of the homogeneous dyadic PS-catalyst system as a benchmark for the Re-PCN assemblies towards CO_2_ reduction. To verify the dyadic system in solution, UV-vis spectra of 0.05 mM 2-Zn in MeCN before and after the addition of equimolar quantities of 1 were recorded (Fig. S14†). These displayed a 5 nm bathochromic shift of the Soret band from 414 to 419 nm indicating self-assembly of 1 and 2-Zn in solution.^41^

**Fig. 3:**
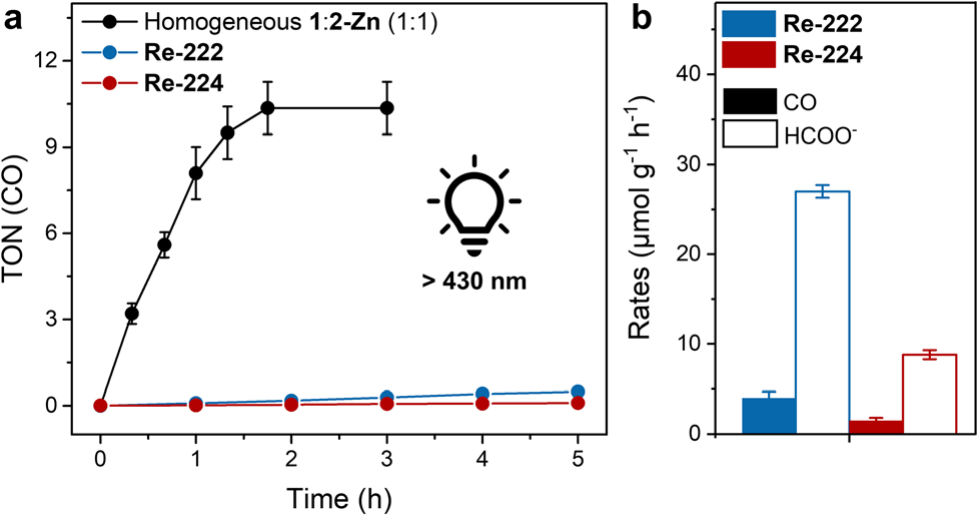
Photocatalytic CO_2_ reduction performance. (a) TON(CO) *vs.* time plot for MOF samples, including the homogeneous reference (black trace). (b) Product evolution rates averaged over the first two irradiation hours. Standard conditions: MOF sample (1.5 mg) suspended in MeCN (4 mL) and deionised H_2_O (0.12 mL) with BIH (225 mg), room temperature, continuous >430 nm irradiation.

As ReX(CO)_3_(bpy)-based catalysts can photoreduce CO_2_ without an external photosensitiser,^22,42^ irradiation of 1 in the catalytic media (identical to heterogeneous conditions above) gave selective CO formation with a maximal TON_CO_ of ∼5 after 2 h. Repeating the experiments in presence of an equimolar amount of 2-Zn yielded TON_CO_ ∼10 (Fig. 3a and Table S2, see ESI† for TON calculation). While low stability under photocatalytic conditions is a common issue of Re catalysts, such a low activity compared to other reports on ReX(CO)_3_(bpy)-based catalysts and porphyrin–rhenium-catalyst dyads (with TONs in the low 100s) highlights the influence of the (pyridine) linking units.^22,26–28,43^

Remarkably, irradiating Re-222 colloids in photocatalytic media for 80 h conversely produced ∼27 μmol_HCOO^−^_ g^−1^ h^−1^ (TON_HCOO^−^_ per node ∼5) and subcatalytic amounts of CO (∼4 μmol_CO_ g^−1^ h^−1^; TON_CO_ per Re < 1) (Fig. 3a, b, Tables S3 and S4†). Incident photon conversion efficiency measurements and apparent quantum yield (AQY) calculations (details in ESI, Tables S5 and S6†) gave values of ∼0.01% (CO) and ∼0.10% (formate) at *λ* = 450 nm. AQYs in the range of 0.1–3.0% are common for colloidal visible light-driven CO_2_ reduction for comparable systems.^19,44,45^ Control photocatalysis experiments using Re-free 222-Zn showed no CO evolution but a comparable formate production rate, while experiments run without BIH or light yielded in all cases no detectable CO, H_2_ or formate (Table S3†). Product generation can be ascribed to light absorption by 2 followed by electron transfers to the CO-selective Re catalysts or to the Zr-oxo node-catalysts for formate production. The latter is in line with previous reports, and suggest two competing CO_2_ reduction mechanisms concomitantly occurring in Re-222.^17,18,39,46^ The corresponding Re-224-based experiments showed overall lower TONs and rates than for Re-222 but similar reactivity trends favouring formate over CO production, *i.e.*, ∼2 μmol_CO_ g^−1^ h^−1^ and ∼9 μmol_HCOO^−^_ g^−1^ h^−1^ (Fig. 3a, b and Table S3†).

While interfacing the Re catalyst amid the MOF significantly increases its lifetime compared to when employed in homogenous conditions, its catalytic involvement is limited as formate evolution prevails. Together with impeded CO evolution, differing numerical performance between the two hybrid MOFs underlines a key influence of the host on the photocatalytic activity.

Post-catalysis PXRD analysis of Re-222 and Re-224 showed crystalline frameworks, while the Re(CO)_3_ moiety was no longer visible in the corresponding ATR-IR spectrum (Fig. S15 and S16†). This suggests that while the host is virtually stable under catalysis conditions, the catalyst is not, in line with its homogeneous instability.^9,43,47^

To examine the reasons for the differing product evolution rates in Re-222 and Re-224 samples, as well as the dramatic change of product selectivity between homogeneous and heterogeneous systems, the photocatalytic mechanism was investigated through spectroscopic and catalytic experiments.

### Catalytic mechanism investigation and photocatalysis under selective irradiation

**Fig. 4:**
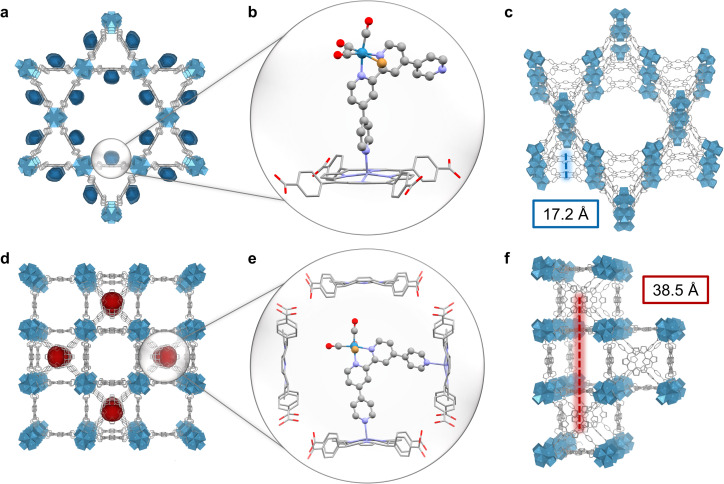
(a) DED map for Re-222. (b) DFT-optimised dyad of 1 and 2-Zn in 222's pore fragment. Red: oxygen, grey: carbon, purple: N, gold: Br, cyan: Re. H atoms omitted for clarity. (c) Translational exciton hopping distance in 222-based assemblies. (d) DED map for Re-224. (e) DFT-optimised dyad of 1 and 2-Zn in 224's pore fragment. (f) Translational exciton hopping distance in 224-based assemblies.

The topology of PCN-222 and -224 differs significantly. While 222 has hexagonal channels of ∼35 Å in size and trigonal channels of ∼10 Å, 224 has cubic pores/impeded channels with ∼20 Å diameter (Fig. 1c and S10†).^8,13^ The geometric structure of the catalyst optimised by DFT calculations yielded a maximum van-der-Waals sphere diameter of ∼13.5 Å, smaller than the MOFs' pore apertures (Fig. 1b, atomic coordinates in ESI†). Despite similar molecular loadings of 1, partial pore blocking is likely more prominent in Re-224 than Re-222 as shown by the former's lower N_2_ uptake (Fig. 2c and Table S1†). DED analysis was thus performed to map the electron density introduced by 1. Briefly, measured PXRD patterns were Le Bail-refined and the resulting structure factors were used to create structure envelopes for pristine and loaded MOFs, which were subtracted from each other (Fig. S17–S20, details in ESI†).^48^ For Re-222, 1 was found directed into the hexagonal channels leaving inner space for substrate diffusion (Fig. 4). In contrast, for Re-224, 1 fills almost the complete space of every second pore. These observations are supported by DFT calculations of 1 in a pore fragment of each MOF, confirming the catalyst positions suggested by DED analysis (Fig. 4a, b, d and e; details and atomic coordinates in ESI†). Specifically, for Re-224, this implies pore blocking potentially further rationalised by DFT calculations that also revealed the possibility of anchoring 1*via* two coordinating N–Zn bonds to neighbouring Zn-porphyrins in Re-224. Together with ATR-IR spectra (shift of Zn–N bands after 1 loading), steric pore restrictions (particularly for 224-Zn), and comparable catalyst loadings per linker for Re-222/Re-224, the DED analysis supports that anchoring of 1 occurs predominantly at the TCPP-bound Zn (Fig. 4, S5 and Table S1†). While not directly observed, partial coordination to physisorbed Zn cannot be ruled out.

Overall, these analyses depict a more hindered nano-environment in Re-224 than in Re-222 potentially limiting reactant (*e.g.*, BIH max. diameter = 10.6 Å),^21^ and product diffusion in line with a lower catalytic activity for the former.

Besides mass transport, transferring conclusions from recent reports on MOF-based exciton migration suggest that charge separation (CS) probability is significantly favoured for Re-222 over Re-224, due to topology-induced higher exciton hopping rates and a lower Förster radius R between chromophores (Förster resonance energy transfer scaling with R^−6^).^20,49^ Here, the translational hopping distance along the *c*-axis is ∼17 Å for 222, and ∼39 Å for 224 (Fig. 4c and f and non-translational distances in Fig. S21†). Additionally, 222's triangular motif with cofacial conformation translating along the lateral direction of the crystallographic *c*-axis further improves excited state delocalisation and hopping rates,^50^ due to head-to-tail coupling.^51^

**Fig. 5:**
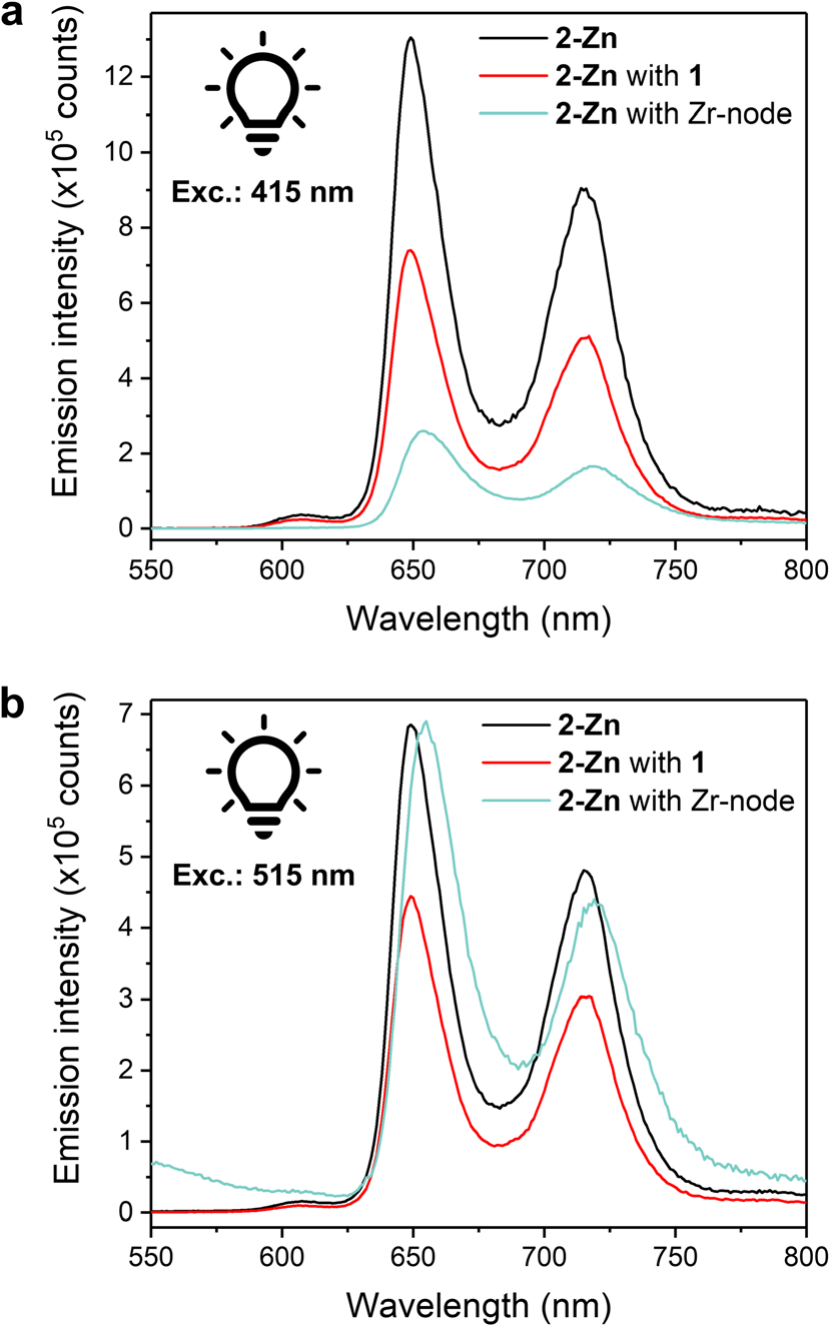
Liquid-phase fluorescence spectra of MeCN/DMF (40/1 v/v) solutions containing 2-Zn (0.05 mM), 2-Zn (0.05 mM) and 1 (0.05 mM), and 2-Zn (0.05 mM) with the isolated Zr_6_-oxo-node (0.2 mM). (a) Excitation at 415 nm. (b) Excitation at 515 nm.

Steady-state luminescence measurements were conducted towards further understanding of photoinduced processes in our systems. Luminescence experiments on a 0.05 mM solution of 2-Zn in MeCN showed two prominent emission bands at *λ* = 649 and 714 nm upon excitation at either 415 or 515 nm (Soret or Q band, respectively) (Fig. 5a and b), matching literature reports ascribed to the de-excitation of the S_1_ states.^52^ The latter is produced by: internal conversion of the S_2_ state, *via* Soret region irradiation, or directly, *via* Q-band irradiation. Adding an equimolar amount of 1 to the solution to replicate homogeneous catalysis system resulted in a quenching of the luminescence intensity, comparable for both excitation wavelengths, and ascribed to photoinduced intramolecular electron transfers from 2-Zn to 1. Incomplete quenching possibly stems from dynamic self-assembly and unpaired 2-Zn as well as inefficient electron transfer. With fluorescence experiments on colloid MOFs being challenging due to high scattering and sedimentation propensity, fully soluble Zr-oxo-based assemblies were prepared as small-scale counterparts of the hybrid PCN systems to enable properties replication and photophysical investigations. Discrete Zr_6_-oxo-nodes (Zr_6_), featuring methacrylate ligands as capping agents to prevent aggregation, were synthesised and isolated following a literature procedure (see ESI†).^53^ Associating these with 2-Zn yielded the fully soluble CO_2_H-tethered composites Zr_6_|2-Zn (see ESI† for assembly conditions) and allowed solution fluorescence measurements without affecting 2-Zn's absorption properties (Fig. S22†). In stark contrast to the Zr_6_-free homogeneous systems, a lower luminescence intensity was recorded after excitation at 415 nm (Fig. 5a) whereas excitation at 515 nm afforded a spectrum comparable to the one of 2-Zn (Fig. 5b). These behaviours were also reproduced in the corresponding action spectra (*λ*_em_ = 720 nm) as Zr_6_|2-Zn displayed unchanged Q-band contributions compared to 2-Zn, and adding 1 resulted in lesser contributions (Fig. S23†).

Such wavelength-dependent luminescence behaviour hints at different energy relaxation pathways. Here, excitation into the Soret band in the Zr_6_|2-Zn assembly results in a high-energy S_2_ state (zero–zero transition energy, *E*_00_ ≈ 2.9 eV) and likely induces fast electron transfer to a Zr-oxo cluster.^18^ As no fluorescence quenching was observed upon Q-band irradiation, this pathway is disabled from the S_1_ state possibly due to its lower energy level (*E*_00_ ≈ 2.0 eV). This also implies that the kinetics of charge separation from S_2_ are significantly faster than the S_2_ → S_1_ internal conversion. While Zn porphyrins present various and complex relaxation mechanisms, this interpretation is coherent with the documented slow S_2_ → S_1_ kinetics in Zn porphyrins, and in line with hot electron transfers from S_2_ as previously observed in molecular dyads and in heterogeneous assemblies.^25,54,55^ As inferred by the wavelength-independent quenching of fluorescence in the 2-Zn with 1 system (Fig. 5), reduction of 1 from the S_1_ excited state of 2-Zn is anticipated and consistent with the significant associated exergonicity (change in Gibbs free energy value, Δ*G*_CS_ = −0.6 eV).

To investigate whether this behaviour is reflected in the photocatalysis performance, we performed a series of wavelength-dependent investigations using lower energy-centred wavelengths *λ* = 490–740 nm (∼8 mW cm^−2^), thus omitting Soret band excitation and corresponding S_2_ excited state production.

**Fig. 6:**
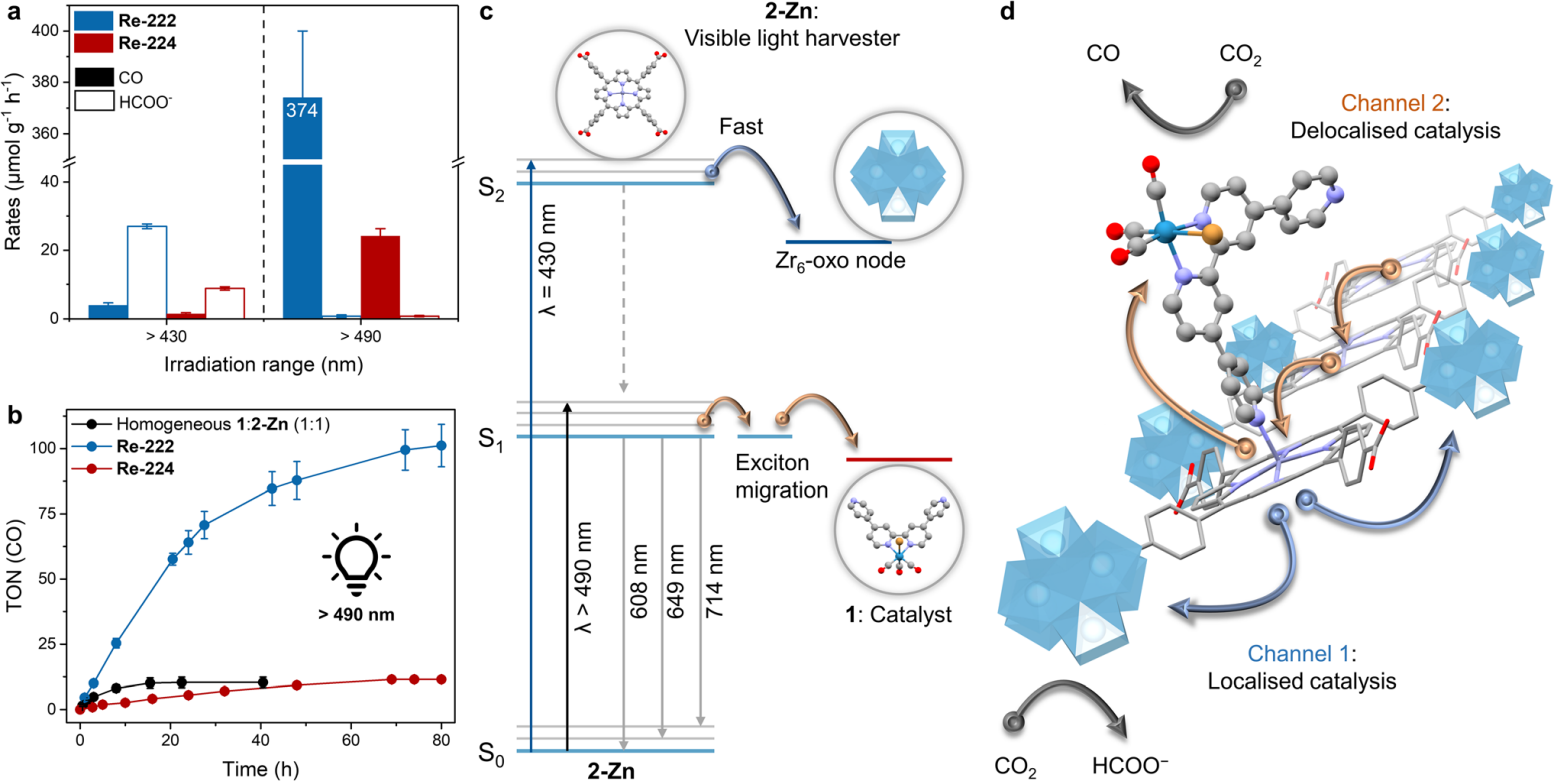
Wavelength-dependent photocatalytic CO_2_ reduction with Re-PCN assemblies. (a) Product evolution rates depending on irradiation wavelength averaged over the first two irradiation hours. (b) TON(CO) *vs.* time plot for MOF samples irradiated >490 nm. Includes the homogeneous reference (black trace). Standard conditions are MOF sample (1.5 mg) suspended in MeCN (4 mL) and deionised H_2_O (0.12 mL) with BIH (225 mg), room temperature, continuous irradiation. (c) Schematic energy level diagram illustrating the working principle upon irradiation at different wavelengths. (d) Schematic representation of the two irradiation wavelength–dependent electron channels observed in Re-PCN hybrids to either deliver localised electrons to the node for CO_2_-to-formate, or delocalised exciton migration followed by charge separation to the molecular catalyst for CO_2_-to-CO. Note that for reasons of clarity not all nodes are shown.

For Re-222-based experiments, a dramatically increased reactivity, and a reversed selectivity from HCOO^−^ to CO were observed with ∼370 μmol_CO_ g^−1^ h^−1^ and <1 μmol_HCOO^−^_ g^−1^ h^−1^ (Fig. 6a and Table S3†). Correspondingly, TON_CO_ ∼100 were reached after 80 h compared to below 1 obtained with *λ* > 430 nm and a higher absolute light intensity (Fig. 3a, 6b, Tables S3 and S4†). Re-224-based experiments gave a similar trend with ∼24 μmol_CO_ g^−1^ h^−1^ (TONs_CO_ ∼ 12) and <1 μmol_HCOO^−^_ g^−1^ h^−1^ after ∼60 h, albeit with the absolute performance remaining significantly inferior to Re-222 due to its topological limitations (Fig. 6a, b and Table S3†). With selective irradiation predominantly yielding formate or CO (Table S3†), electronic communication between nodes and Re catalysts within the pores is unlikely. After 80 h of >490 nm irradiation PXRD analysis of Re-222 and Re-224 showed crystalline framework retention, while the Re(CO)_3_ moiety was no longer visible in ATR-IR spectra, comparable to >430 nm irradiation (Fig. S15 and S16†). For Re-222 and Re-224, post-catalysis ICP-MS measurements revealed that 27 and 17% of immobilised Re leached into the supernatant, respectively (Table S7†). This is consistent with other reported photodegradations of Re catalysts and described complex decomposition and subsequent loss of activity.^9,43,47,56^ In addition, corresponding dynamic light scattering measurements revealed no Re nanoparticle formation (Fig. S24†).

Control experiments with ^13^C-labelled CO_2_ for both Re-PCN hybrids produced only ^13^CO, confirming that CO_2_ was the sole source of CO (Fig. S25†). Further control experiments conducted with the CO_2_H–analogue of 1, *i.e.*, *fac*-ReBr(CO)_3_(4,4′-dicarboxy-2,2′-bipyridine), node-anchored in PCN-222 and PCN-224 (synthesis in ESI, Fig. S26–S28 and Table S1†) showed comparable wavelength-dependent performance trends to Re-222 and Re-224 under identical reaction conditions (Table S3†). This is consistent with a previously reported PCN-222-Re assembly displaying selective CO_2_-to-CO evolution upon >500 nm irradiation^19^ and thus excludes the catalyst and dyadic motif as the source of the observed trends.

These wavelength-dependent results suggest that excited state (S_2_*vs.* S_1_)-specific, selectivity-dictating catalytic channels are occurring in the MOF assemblies, with electron delivery to the CO evolution molecular catalyst or the formate producing node being selectively activated by low or high energy wavelength, respectively. This was confirmed by AQY measurements at *λ* = 520 nm, giving ∼0.35% (CO) and ∼0.01% (formate), showing a reverse trend compared to 450 nm values (Tables S5 and S6†).

Interestingly, the correlations between product selectivity and irradiation wavelength were found in both Re-PCN assemblies as well as in the homogeneous Zr_6_|2-Zn|1 system. The latter showed limited CO evolution (TONs ∼ 1) and traces of formate using full irradiation, while >490 nm irradiation experiments yielded TONs ∼10 (Table S2†). By contrast, homogeneous Zr_6_-free molecular 2-Zn with 1 delivered wavelength-independent performance. This further highlights the critical impact of the assembly/framework (MOF *vs.* molecular assemblies) on photocatalysis (Fig. 3a, b, 6a, b, Tables S2 and S3†).

Luminescence and catalytic experiments together provide consistent evidence that irradiation with *λ* > 430 nm during photocatalysis mainly results in excitation of the Soret-band (S_0_–S_2_ transition, Fig. S6–S8†) in PCN assemblies upon which the S_2_ state is rapidly quenched by electron transfer to the Zr_6_-oxo-node (Fig. 6c).^17,18,39^ Here, bimolecular reaction with the diffusing SED is likely to proceed subsequently to regenerate the ground state (Fig. S13†) or detrimental charge recombination occurs.^19^ Selective CO_2_-to-formate reduction proceeds *via* reduced Zr(iii) centres upon collecting another electron.^17,46^ With each linker connected to four nodes and charge separation occurring at the light harvesting centre, this constitutes a localised electron channel (Fig. 6d). Shifting to irradiation with *λ* > 490 nm mainly results in excitation of the Q bands (S_0_–S_1_ transition),^55^ affording a lower energy excited state unable to trigger node-quenching but able to reduce 1. The latter effects selective CO_2_-to-CO reduction upon accumulating two electrons.^9,19^ Alternatively, in absence of a Re catalyst in direct vicinity, directional exciton migration proceeds within the MOF structure.^19^ As an exciton can visit 100+ linkers in its lifetime,^19,51^ this energy funnelling is highly delocalised and provides a long-range, antennae-like catalytic channel (Fig. 6d).

The minute, deactivated CO evolution when using full irradiation is noteworthy considering that Soret and Q bands are concomitantly excited. The rationale is likely intricated and may involve substrate diffusion, competing photophysical processes and slower CO catalysis kinetics. Tentatively, the high molar absorption of S_2_ together with an excess of nodes to catalysts (statistically each linker neighbours four Zr_6_ nodes and ∼0.1 Re catalysts, Table S1†) could result in a kinetic preference for available CO_2_ (with affinity to coordinate to Lewis–acidic Zr nodes) to be converted to formate, depleting local substrate concentrations.^57^ While future in-depth spectroscopic studies are needed to elucidate this phenomenon, this finding may hold important repercussions as most literature research focuses on full spectrum broadband irradiation.

Overall, the results show that the linker's excited states and quenching kinetics with the molecular catalyst *vs.* the node are critical for understanding and tailoring reactivity. As a perspective, the observed wavelength-dependency provides an edge over similar systems as these usually show a reverse trend, *i.e.*, high-energy (near) UV irradiation achieves higher performance.^45,58^ Here, lower-energy visible light provides selective molecular CO_2_-to-CO reduction, allowing unusual control over product selectivity based on irradiation wavelength.

## Experimental

For a comprehensive description of analytical methods and experimental procedures the reader is referred to the ESI.† An overview of the central syntheses is provided here.

### Synthetic procedures

#### 4,4′:2′,2′′:4′′,4′′′-Quarterpyridine (qtpy)

The synthesis was adapted from a literature known procedure.^30^ 4,4′-Bipyridine (2.50 g, 16.0 mmol) and 0.50 g Pd/C (10 wt%) were heated in a Teflon bomb at 250 °C for 48 h. The resulting solid was ground and Soxhlet extracted with dichloromethane (DCM, 300 mL) over 18 h, giving a first fraction. The Pd residue was washed with hot DMF (2 × 50 mL), giving a second fraction. The solvents of both fractions were removed, the residues were combined, and purified by flash column chromatography (DCM/MeOH 91/9 v/v) and subsequently *via* sublimation to yield the pure product (0.77 g, 31%), whose characterisations matched literature reports (see ESI†).

#### *fac*-ReBr(CO)_3_(qtpy) (1)

The synthesis was adapted from a literature known procedure.^31^ ReBr(CO)_5_ (140 mg, 0.35 mmol, 1.00 eq.) was dissolved in a mixture of dry and degassed toluene/THF (25 mL, 3/1 v/v) under Schlenk conditions. Subsequently, 4,4′:2′,2′′:4,4′′′-quarterpyridine (112 mg, 0.36 mmol, 1.05 eq.) was added. The yellow solution was refluxed for 24 h. After cooling, an orange solid precipitated. The suspension was filtered and the residue washed with CHCl_3_ (2 mL). The solid was dried *in vacuo* to give the pure product as an orange powder (144 mg, 63%), whose characterisations matched literature reports (see ESI†).

#### 5,10,15,20-Tetrakis(4-carboxylphenyl)-porphyrin (2)

The synthesis was adapted from a literature known procedure.^32^ The first step was the synthesis of 5,10,15,20-tetrakis(4-methoxycarbonylphenyl)-porphyrin (TPPCOOMe) by adding pyrrole (3.09 mL, 44.9 mmol, 1.1 eq.) and methyl-*p*-formylbenzoate (6.93 g, 42.3 mmol, 1.0 eq.) to refluxing propionic acid (100 mL). After refluxing for 22 h under continuous stirring, the precipitate was filtrated and washed with MeOH (30 mL), EtOAc (10 mL) and THF (10 mL) to yield the purple product (5.45 g, 6.44 mmol, 15% yield), whose characterisations matched literature reports (see ESI†). In the second step, TPPCOOMe (1.00 g, 1.19 mmol, 1.0 eq.) was dissolved in a 1 : 1 mixture of THF/MeOH (70 mL). A solution of KOH (3.5 g, 62.4 mmol, 52 eq.) in H_2_O (30 mL) was added. The resulting mixture was refluxed for 15 h. After removing the organic solvents *in vacuo*, the solid was redissolved in H_2_O (150 mL) at 90 °C for 15 min. The solution was filtered and acidified with 1 M HCl (100 mL). The resulting green precipitate was filtered and dried to obtain the product (864 mg, 1.09 mmol, 92% yield), whose characterisations matched literature reports (see ESI†).

#### 5,10,15,20-Tetrakis(4-carboxylphenyl)-porphyrin-Zn(ii) (2-Zn)

TPPCOOMe (854 mg, 1.01 mmol, 1.0 eq.) and ZnCl_2_ (1.75 g, 12.8 mmol, 12.8 eq.) were refluxed in DMF (100 mL) for 6 h. After the mixture was cooled to room temperature, H_2_O (150 mL) was added. The dark purple precipitate was filtered and washed with H_2_O (2 × 50 m mL). The obtained solid was dissolved in CHCl_3_ (500 mL) and washed with H_2_O (3 × 250 mL). The organic layer was dried over anhydrous Na_2_SO_4_. Subsequently, the solvent was removed by rotary evaporation to yield TPPCOOMe-Zn(ii) (276 mg, 303 μmol, 30% yield, see ESI†).

This intermediate (214 mg, 235 μmol, 1.0 eq.) was dissolved in THF/MeOH 1 : 1 (15 mL). KOH (691 mg, 12.3 mmol, 52 eq.) was dissolved in H_2_O (6 mL) and added to the prior solution. The reaction mixture was refluxed for 5 h under continuous stirring. After the mixture was cooled to room temperature, the organic solvents were removed by rotary evaporation. The crude product was dissolved in H_2_O (32 mL) and heated at 90 °C for 10 min. After cooling to room temperature, the aqueous mixture was acidified with 1 M HCl solution (21 mL) and 37% HCl (1 mL) in respective order. The resulting dark green precipitate was isolated by centrifugation and washed with H_2_O (8 × 30 mL). The dark green product was dried *in vacuo* overnight (137 mg, 161 μmol, 68% yield, see ESI†).

#### PCN-222

The synthesis was adapted from a literature known procedure.^14,33^ In a 20 mL screw cap vial, 2 (12.5 mg, 0.016 mmol, 1.00 eq.) and ZrOCl_2_·8H_2_O (23.5 mg, 0.073 mmol, 4.56 eq.) were dissolved in *N*,*N*-diethylformamide (DEF, 3 mL). After addition of 4-*tert*-butylbenzoic acid (1350 mg, 7.57 mmol, 473 eq.), the mixture was ultrasonicated for 10 min. The mixture was heated at 120 °C for 12 h in an oven. The solid was separated by centrifugation and soaked with DMF (3 × 6 mL) and dried *in vacuo*. The solid (12.2 mg) was dispersed in DMF (8.1 mL) and acidified with 8 M HCl (0.3 mL). The mixture was heated in an oven at 120 °C for 12 h. The purple solid was collected by centrifugation and soaked in DMF (3 × 6 mL) and acetone (3 × 6 mL) and dried *in vacuo* to yield the product, whose characterisations matched literature reports (see ESI†).

#### PCN-224

The synthesis was adapted from a literature known procedure.^14,33^ In a 20 mL screw cap vial, 2 (16.0 mg, 0.020 mmol, 1.00 eq.) and ZrOCl_2_·8H_2_O (30.0 mg, 0.093 mmol, 4.65 eq.) were dissolved in DMF (4 mL). After addition of formic acid (0.69 mL, 17.2 mmol, 860 eq.) and 3,3-dimethylbutanoic acid (0.81 mL, 6.36 mmol, 318 eq.), the mixture was ultrasonicated for 10 min. The mixture was heated at 120 °C for 12 h in an oven. The solid was separated by centrifugation and soaked with DMF (3 × 6 mL) and dried *in vacuo*. The solid (31.1 mg) was dispersed in DMF (21 mL) and acidified with 8 M HCl (1.6 mL). The mixture was heated in an oven at 120 °C for 12 h. The purple solid was collected by centrifugation and soaked in DMF (3 × 10 mL) and acetone (3 × 10 mL) and dried *in vacuo* to yield the product, whose characterisations matched literature reports (see ESI†).

#### Metalation of 222 or 224 with Zn

The synthesis was adapted from a literature known procedure.^34,35^ ZnCl_2_ (59.9 mg for 222 and 22.9 mg for 224) was dissolved in DMF (3 mL). After addition of the MOF powder (30 mg), the suspension was heated in an oven at 100 °C for 24 °C. The product was isolated by centrifugation and washed with DMF (3 × 6 mL) and acetone (3 × 6 mL). Drying *in vacuo* yielded the product, whose characterisations matched literature reports (see ESI†).

#### Molecular catalyst loading in 222-Zn or 224-Zn

A 0.1 mM solution of 1 in MeCN (15 mL) was added to powder samples of the respective activated MOF sample (10.0 mg) in VWR® TraceClean® 20 mL vials. After 24 h in the dark, the suspension was centrifuged, and the supernatant removed. The resulting powder was washed with fresh MeCN (3 × 7 mL) for 2 h per wash cycle and then dried overnight *in vacuo*.

#### Photocatalytic CO_2_ reduction

Photocatalytic tests were performed in air-tight 50 mL Schlenk flasks with the respective MOF material (1.5 mg), a stir bar, MeCN (4 mL), deionised H_2_O (0.12 mL, 3 vol%) and BIH (225 mg, 1 mmol). For CO_2_ reduction the reaction suspension and headspace were fully saturated with CO_2_ for seven minutes and after sealing a CO_2_ overpressure was applied, totalling a pressure of 1.45 bar.

The irradiation source was a heat-free white light generator Asahi Spectra MAX-303 Compact Xenon Light Source 300 W, with either a XVL0430-Longpass 430 nm filter (430–740 nm irradiation, ∼10 mW cm^−2^) or a XVL0490-Longpass 490 nm filter (490–740 nm irradiation, ∼8 mW cm^−2^). Reaction products were determined through headspace analysis by gas chromatography and ^1^H NMR of the reaction solution.

## Conclusions

We successfully synthesised and characterised two different hybrids of *fac*-ReBr(CO)_3_(qtpy) in porphyrinic MOFs (PCN-222 and PCN-224) by post-synthetic self-assembly, allowing straightforward topology-independent catalyst anchoring. Corresponding smaller scale, molecular and supramolecular assemblies were also prepared for comparison and benchmarking purposes. Here, we demonstrated that material interfacing at various scales, *i.e.*, molecule–molecule (1 + 2-Zn), molecule-cluster (Zr_6_|2-Zn|1), and molecule-particle (Re-PCNs) produces composites displaying unique and complex behaviours that are concomitantly reminiscent, diverging and unseen in their individual building blocks.

Mainly, assembling Re catalyst-PCN composites afforded photocatalysts bearing two catalytic channels that are selectively activated by specific incident irradiation wavelengths and thus, ultimately govern the catalysis product selectivity. Shorter wavelengths resulted in localised charge transfers from the excited porphyrin linkers to the Zr_6_-oxo-nodes, producing formate. Alternatively, lower energy irradiation can be used to activate the CO-selective Re catalysts with exciton migration enabling long-range, delocalised catalysis. By contrast, the corresponding molecular and supramolecular systems yielded product selective and wavelength-independent catalysis.

While molecule-like localised S_2_ and S_1_ states more accurately describe the observed photoinduced processes in Re-PCNs over typical band-like description of MOFs, the hybrids also display material-like properties elusive to molecular species. Thus, a topology effect was shown with the PCN-222 hybrids displaying a higher photocatalytic activity over PCN-224, as small pore sizes hinder mass diffusion and short coupling distances promote antennae-effect. Further the nanoparticular hybrid porous systems enabled a 10-fold increase in CO_2_ reduction activity compared to their discrete counterparts, ascribed to stabilising pore confinement.

Although preliminary, this work constitutes the seminal investigation of the likely considerable influence of MOF topology on solar fuel production. Future efforts on controlled distances between catalyst and photosensitiser, understanding of underlying photoinduced processes, varied anchor principles, as well as optimised energy and mass transport will allow orthogonal component assembly and minimise energy loss for efficient solar fuel production. Finally, we show that while broadband light is often considered in its entirety in the broader literature, a finer differentiation may be needed to understand complex hybrid materials' behaviour and performance.

## Data availability

The source data is available from the corresponding author upon reasonable request.

## Author contributions

P. M. Stanley: methodology, validation, formal analysis, investigation, data curation, writing – original draft, visualisation. K. Hemmer: methodology, validation, formal analysis, investigation, data curation, writing – original draft, visualisation. M. Hegelmann: investigation, data curation. A. Schulz: investigation, data curation. M. Park: investigation, data curation. M. Elsner: investigation. M. Cokoja: conceptualisation, writing – review and editing, supervision, resources, project administration. J. Warnan: conceptualisation, writing – review and editing, supervision, resources, project administration.

## Conflicts of interest

There are no conflicts to declare.

## Supplementary Material

SC-013-D2SC03097G-s001
