# Supplementary material for: Topology- and wavelength-governed CO2 reduction photocatalysis in molecular catalyst-metal–organic framework assemblies
Source: Chem Sci. 2022 Oct 3;13(41):12164–74. doi: 10.1039/d2sc03097g (PMC9601321; doi:10.1039/d2sc03097g)
Supplement: SC-013-D2SC03097G-s001 [file SC-013-D2SC03097G-s001.pdf]

Electronic Supplementary Material (ESI) for Chemical Science.
This journal is © The Royal Society of Chemistry 2022

Supporting information

Topology- and wavelength-governed CO_2 reduction photocatalysis in molecular catalyst-metal-organic framework assemblies

Philip M. Stanley,[†a] Karina Hemmer,[†a] Markus Hegelmann,[a] Annika Schulz,[a] Mihyun Park,[a] Martin Elsner,[b] Mirza Cokoja,[a] and Julien Warnan*[a]

[†] These authors contributed equally.

[a]*Chair of Inorganic and Metal-Organic Chemistry; Department of Chemistry, TUM School of Natural Sciences, Catalysis Research Center (CRC), Technical University of Munich, Garching, Germany*

[b]*Chair of Analytical Chemistry and Water Chemistry, Department of Chemistry, TUM School of Natural Sciences, Technical University of Munich, Garching, Germany*

**Correspondence:* julien.warnan@tum.de

Table of Contents

1. Analytical methods, calculations, and characterisation techniques S2
2. Synthetical procedures S7
3. Supporting Figures S12
4. Supporting Tables S26
5. Bibliography S32
6. Atomic coordinates for DFT calculated molecules S33
7. SUPERFLIP Input File S41

1. Analytical methods, calculations, and characterisation techniques

Materials

Acetonitrile (MeCN, HPLC grade), methanol (HPLC grade), chloroform (HPLC grade), absolute ethanol, *n*-propanol (HPLC grade), ethyl acetate (HPLC grade), ethylene glycol dimethyl ether, hexane (HPLC grade), tetrahydrofuran (THF, HPLC grade), *N,N*-dimethylformamide (DMF, HPLC grade), dichloromethane (DCM, HPLC grade), $ReBr(CO)_5$, polyphosphoric acid, *o*-phenylenediamine, benzoic acid, methyl iodide, Celite® S, sodium borohydride, anhydrous sodium sulfate, potassium oxalate, $ZrOCl_2 \cdot 8\ H_2O$, pyrrole, propionic acid, 4-tert-butylbenzoic acid, 3,3-dimethylbutanoic acid, Zr(IV) propoxide, formic acid, Pd/C (10 wt. %), sodium hydroxide, and potassium hydroxide were purchased from Sigma Aldrich. Methyl-p-formylbenzoate was purchased from ChemPUR. Diethylformamide, 2,2'-Bipyridine, 4,4'-bipyridine, and 4,4'-dicarboxyl-2,2'-bipyridine were purchased from TCI Chemicals. Methacrylic acid was purchased from ACROS Organics. $FeCl_3$, $ZnCl_2$, and 8-dram vials for MOF synthesis and molecular loading (TraceClean®) were purchased from VWR. All purchased chemicals were used without further purification. Where specified, inert gas conditions entail standard *Schlenk* techniques under an argon atmosphere.

Adsorption measurements

Adsorption measurements with N_2 (99.999 vol%) at 77 K were carried out on a 3Flex Physisorption from *Micromeritics Instrument Corp.*, which uses a volumetric method to determine the amount adsorbed under an equilibrated gas pressure. Adsorption data were processed using the 3Flex Software Version 5.01 by *Micromeritics Instrument Corp.* and plotted in OriginPro 2019b by *OriginLab Corp.* Samples were transferred into pre-weighed sample tubes and capped with *Micromeritics* CheckSeals. Samples were subsequently activated at 393 K for 12 hours under a dynamic vacuum of approx. 10^{-3} mbar using a SmartVac Prep by *Micromeritics Instrument Corp.* to ensure absence of unwanted adsorbates and identical pre-measurement states of all samples. In particular, this unusually low activation temperature and time was chosen for all samples to ensure structural integrity of the immobilised molecular complexes which are known to sublime and decompose comparatively readily. The mass of the adsorbents was then recorded, generally in the range of 25 – 40 mg. The free space of the sample tube was determined prior to measuring each adsorption isotherm using Helium (99.999 vol%). A liquid nitrogen bath was used for measurements at 77 K.

The apparent surface area was derived using the Brunauer-Emmett-Teller (BET) model, is hence given as the 'BET area' and based on N_2 isotherms measured at 77 K. To determine this value for microporous materials, care was taken to adhere to the Rouquerol criteria.[1]

The pore size distribution (PSD) was derived by fitting N_2 isotherms measured at 77 K with sets of theoretical isotherms (kernel) derived from two-dimensional non-local-density functional theory (2D-NLDFT) based methods for specific pore sizes and geometry. As an approximation, cylindrical pores on an oxide surface were assumed for all materials to allow comparability within this series. Fitting was done using the

respective kernel available via the 3Flex Software Version 5.01 by *Micromeritics Instrument Corp.*

Attenuated total reflectance infrared spectroscopy (ATR-IR)

ATR-IR measurements were carried out on a *PerkinElmer* Frontier FT-IR spectrometer featuring an ATR plate with a germanium crystal with a 2 cm^{-1} resolution and 16 accumulated scans.

Apparent quantum yield (AQY) determination

The incident light flux under reaction conditions was measured using a 0.15 M ferrioxalate solution following established literature procedures.[2–5] The actinometer experiments were performed modelling reaction conditions (identical *Schlenk* glassware, distance to LEDs, solution volume, and stir bar). Two solutions were prepared, a 0.15 M potassium ferrioxalate solution (7.37 g $K_3[Fe(C_2O_4)_3] \cdot 3\ H_2O$, 90 mL deionised H_2O, and 10 mL 1.0 N H_2SO_4) and a 0.1% buffered phenanthroline solution (22.5 g sodium acetate, 100 mg phenanthroline in 100 mL 0.5 M H_2SO_4) and stored in amber bottles.

In a typical experiment, two *Schlenk* flasks containing 4 mL of the 0.15 M potassium ferrioxalate solution were prepared. One sample was irradiated for 60.0 s with the LED, while the other was left in the dark as a control. Upon completion of irradiation, 667 µL of the 0.1% buffered phenanthroline solution was added to both of the samples. The samples were then allowed to develop in the dark for another 5 minutes before the absorption of each of the samples was measured with UV-Vis spectroscopy. Using the optical difference of absorption at 510 nm between the irradiated and control (dark) sample and a molar absorption coefficient $\varepsilon_{510\ nm}$ = 11100 $M^{-1}cm^{-1}$, the amount of Fe^{2+} produced during the irradiation can be determined. The quantum yield and potassium ferrioxalate incident light absorption at each applied wavelength, respectively, were taken from established literature procedures.[3–5] The measured absorbance values at 510 nm after 60.0 s irradiation and subsequent AQY calculations with formulas are given in Table S5 and Table S6.

The irradiation intensity of ~1 $mW \cdot cm^{-2}$ was verified using a *Thorlabs* PM100D compact power and energy meter console (calibrated and tested by Thorlabs on December 3, 2021) paired with a *Thorlabs* S401C thermal power sensor head (surface absorber, 0.19 - 20 µm, 1 W, Ø10 mm, sensitivity +/- 3%) (calibrated and tested by Thorlabs on December 8, 2021).

Catalyst/linker ratio calculations

From the exact metal loadings determined by ICP-MS measurements (see Table S1) the weight% of Re and Zn were determined and subtracted from the total mass. Subsequently, using the idealised sum formulas of activated PCN-222 ($Zr_6O_8(OH)_8(TCPP)_2$, M = 2463.82 $g \cdot mol^{-1}$) and PCN-224 ($Zr_6O_{12}(OH)_8(TCPP)_{1.5}$, M = 2063.33 $g \cdot mol^{-1}$), the remaining weight% were weighted by the mass fraction assigned to the linkers in the idealised sum formula (0.6382 for PCN-222 and 0.5730 for PCN-224). The obtained weight% of the total assembly mass stems solely from the TCPP linkers, allowing the mol value to be calculated with the linker's molar mass

(786.21 g·mol^{-1}). The catalyst's mol value was also readily obtained from ICP-MS data (see Table S1), and dividing the two yields the catalyst/linker ratio.

Density functional theory (DFT) optimisations and modelling

The applied molecular complexes were optimised on a wB97xD/LANL2DZ level using GaussView 6.0 and Gaussian 16W. Atomic coordinates of the optimised structures are available in chapter S6. Modelling within the MOF pores was performed by extracting the pore geometry from the respective cif files (PCN-222 CCDC 893545; PCN-224 CCDC 1001133), omitting the node, and rigidifying the linker backbone during DFT optimisations at the wB97xD/LANL2DZ level. Bonds between **1**'s free nitrogen atoms and porphyrin-Zn were not forced.

Difference envelope density (DED) analysis

Difference envelope density modeling and analysis was done in accordance with published procedures.[6] Collected PXRD patterns were analysed by Le Bail refinement with *JANA2006*. The refinement of unit cell parameters was initially started from the axial length of the PCN single crystal. The peak profiles of intensity were fitted with a background shift correction, a pseudo-Voigt profile (weighted sum of a Lorentz and Gaussian function), and the Berar-Baldinozzi correction. $|F_{\mathrm{hkl}}|^2$ and the corresponding (h k l) values were extracted and indexed from the fitting profile of the intensity pattern. Structure envelopes were created with *SUPERFLIP* and **222-Zn** or **224-Zn** was subtracted from the catalyst-loaded samples and visualized in VESTA. For an exemplary *SUPERFLIP* input file (.inflip format) see chapter S7.

Dynamic light scattering (DLS)

DLS measurements are performed in a QS Suprasil 10.00 mm quartz glass cuvette from *Heraeus Quarzglas GmbH* with a *Brookhaven Instruments* Zetasizer Nano Series ZEN 3600 to determine the hydrodynamic radius of the particles in solution.

Fluorescence measurements

Optoelectronic compound characterisation was performed with an *Edinburgh Instruments* FS5 spectrofluorometer with a 150 W CW ozone-free xenon arc lamp, Czerny-Turner design monochromators, a R928P photomultiplier emission detector, a Tcspc module and the mcs (phosphorescent) option.

Inductively coupled plasma mass spectrometry (ICP-MS)

To obtain an accurate quantification of the Re and Zn amount immobilised in MOF samples and in post-catalysis supernatants, ICP-MS for rhenium and zinc, respectively, was conducted on a *Perkin Elmer* NexIon 350D ICP-MS instrument. The respective samples were immersed in concentrated 7.5 mL nitric acid and 2.5 mL H_2O_2 30 % (v/v) and treated in the microwave at 150 °C for 5 min. Subsequently, the microwave-digested samples were diluted 1/100 with Millipore Milli-Q® water. Each solvent was extra pure and checked for possible analyte contaminations before measurement. ^{66}Zn and ^{187}Re were used as target masses for the analytes and ^{103}Rh as an internal standard. Analyte quantification was carried out in standard mode with correction equation to avoid polyatomic interferences. Detection limit for Zn was 1.42 µg L^{-1} and 0.10 µg L^{-1} for Re. External Calibration was performed in the range of

0 µg L^{-1} to 100 µg L^{-1}. Each sample was measured with five measurement replicates, a dwell time per 50 ms and an integration time of 750 ms. The Zn and Re concentrations were blank corrected via measurement of blank samples. External calibration was performed in the range of 0 µg L^{-1} to 100 µg L^{-1}.

Infrared spectroscopy (Gas phase IR)

Gas phase IR spectra for isotope labelling studies were recorded with a *ThermoScientific* Nicolet 380-FT and the *OMNICTM* Spectra Software. After a given reaction time, 20 mL of the reaction headspace were transferred to an evacuated gas IR cell (20 cm path length, KBr windows) and a high-resolution transmission spectrum was collected (512 scans, 0.241 cm^{-1} data spacing).

Gas chromatography (GC)

Photocatalytic reaction analysis was conducted via measuring the gas composition of the reaction headspace. For CO/CO_2 this was done with a *Varian* Micro 490-GC, with a CO_x 1m heated column at 80 °C column temperature, 140 kPa column pressure, auto detector sensitivity, 10 s sampling time, and 330 s run time. These parameters provide complete and quantitative separation of nitrogen/air, CO, and CO_2 in the sample. The absence of H_2 was verified with a *SRI Instruments Europe* Model 8610C, with a reducing gas detector (RGD), a MoleSieve 13X and a silica gel column with N_2 as the carrier gas was used. After sample injection onto the column at 52 °C the temperature was held for 8 minutes. CO concentrations were calibrated with several reference ppm gases from *Air Products GmbH* for accurate quantification.

Nuclear magnetic resonance (NMR)

Liquid state NMR spectra were recorded by a *Bruker* Ultrashield DRX400 (^{1}H: 400.13 MHz) at ambient temperature (298 K). The ^{1}H NMR spectroscopic chemical shifts δ are reported in ppm relative to tetramethylsilane. ^{1}H NMR spectra are calibrated against the residual proton and natural abundance carbon resonances of the respective deuterated solvent as an internal standard (CD_3CN: δ (^{1}H) = 2.15 ppm, DMSO-d_6: δ (^{1}H) = 2.50 ppm). The following abbreviations are used to describe signal multiplicities: s = singlet, d = doublet, t = triplet, m = multiplet.

Photocatalytic reactions

Photocatalytic tests were performed in air-tight 50 mL *Schlenk* flasks with the respective MOF material (1.5 mg), a stir bar, acetonitrile (4 mL), deionised H_2O (0.12 mL, 3 vol%) and BIH (225 mg, 1 mmol). For CO_2 reduction the reaction suspension and headspace were fully saturated with CO_2 for seven minutes, and after sealing, a CO_2 overpressure was applied, totalling a pressure of 1.45 bar.

The irradiation source was a heat-free white light generator *Asahi Spectra* MAX-303 Compact Xenon Light Source 300 W, with either a XVL0430-Longpass 430 nm filter (430-740 nm irradiation, ~10 mW·cm^{-2}) or a XVL0490-Longpass 490 nm filter (490-740 nm irradiation, ~8 mW·cm^{-2}). Reaction products were determined through headspace analysis by gas chromatography and ^{1}H NMR of the reaction solution.

For photocatalysis or luminescence measurements with the model homogeneous Zr_6|**2-Zn**|**1** system, a 4-fold molar $Zr_6(OH)_4O_4(OMc)_{12}$ excess (see synthesis below)

was used to avoid porphyrin aggregation on the same node. The solution was obtained by mixing the three stock solutions and leaving them to assemble for 1 h at room temperature before either conducting photocatalysis (following the standard procedure detailed above) or luminescence measurements.

Photocatalysis Turnover Number (TON) Calculations

For photocatalysis, the TONs were calculated as follows. The exact molar catalyst amount was obtained through multiplying the nmol/mg_{MOF} values from ICP-MS analysis with the weigh-in. Subsequently, the GC headspace analysis provided results for each gas present, calibrated with reference gases as described above. For carbon monoxide these were then converted to moles. To account for solvated CO in MeCN, Henry's law was used and the formulas, values, and an exemplary TON calculation is provided in Table S4.[7,8]

Powder x-ray diffraction (PXRD)

PXRD measurements were performed on a silicon single-crystal wafer using *Bragg-Brentano* geometry in a *Rigaku* MiniFlex 600-C diffractometer. X-ray Cu K_α radiation (λ_1 - 1.5406 Å, λ_2 - 1.5444 Å, I_2/I_1 - 0.5) was used, and K_β radiation was removed by a Ni-filter. The measurement range, unless stated otherwise, was from 2.0° to 50.0° (2θ) with a step size of 0.010 degrees and a scan rate of 5 degrees per minute.

Scanning electron microscopy (SEM)

SEM images were obtained with a *Jeol* JSM-7500F field emission scanning electron microscope with the Gentle Beam mode.

Solid-state UV-Vis spectroscopy

Solid-state UV-Vis spectra were recorded on a *Shimadzu* UV-3600 Plus UV-Vis-NIR spectrophotometer. Powder samples were fixed between two quartz glass slides for measurement. Measurement parameters: Medium Scan Speed, slit width (20) with External(3Detector) unit, enabled stair correction, baseline correction ($BaSO_4$ background), S/R exchange normal, slit and detector lock normal.

UV-Vis spectroscopy

UV-Vis spectra were recorded on an *Agilent Technologies* Cary 60 with a scan rate of 600 nm/min. Baseline correction was performed with the respective pure solvent. Each sample was measured in a QS Suprasil 10.00 mm quartz glass cuvette from *Heraeus Quarzglas GmbH.*

2. Synthetical procedures

4,4':2',2'':4'',4'''-quarterpyridine (qtpy)

The synthesis was adapted from a literature known procedure.[9] 4,4'-Bipyridine (2.50 g, 16.0 mmol) and 0.50 g Pd/C (10 wt. %) were heated in a Teflon bomb at 250 °C for 48 h. The resulting solid was ground and Soxhlet extracted with dichloromethane (300 mL) over 18 h, giving a first fraction. The Pd residue was washed with hot DMF (2 × 50 mL), giving a second fraction. The solvents of both fractions were removed, the residues were combined, and purified by flash column chromatography (DCM/MeOH 91/9 v/v) and subsequently via sublimation to yield the pure product (0.77 g, 31% yield). Analytics matched literature reports.

^{1}H NMR (400 MHz, 300 K, $CDCl_3$) δ (ppm) = 8.84 (dd, 2H), 8.81 (dd, 2H), 8.79 (m, 4H), 7.74 (m, 4H), 7.62 (dd, 2H).

***fac*-ReBr(CO)$_3$(qtpy) (1)**

The synthesis was adapted from a literature known procedure.[10] ReBr(CO)$_5$ (140 mg, 0.35 mmol, 1.00 eq.) was dissolved in a mixture of dry and degassed toluene/THF (25 mL, 3/1 v/v) under *Schlenk* conditions. Subsequently, 4,4':2',2'':4,4'''-quarterpyridine (112 mg, 0.36 mmol, 1.05 eq.) was added. The yellow solution was refluxed for 24 h. After cooling, an orange solid precipitated. The suspension was filtered, and the residue was washed with $CHCl_3$ (2 mL). The solid was dried in vacuo to give the pure product as an orange powder (144 mg, 63 %).

^{1}H NMR (400 MHz, 300 K, DMSO-d$_6$) δ (ppm) = 9.34 (s, 2H), 9.17 (d, 2H), 8.88 (d, 4H), 8.17 (d, 2H), 8.07 (d, 4H)

ATR-IR: 2019, 1929, 1886, 1615, 1592, 1536, 1476, 1402, 1332, 1233, 1070, and 816 cm^{-1}

***fac*-ReBr(CO)$_3$(dcbpy)** (control experiments, dcbpy = 4,4'-dicarboxy-2,2'-bipyridine)

The synthesis was adapted from a literature known procedure.[11] ReBr(CO)$_5$ (0.30 g, 0.74 mmol, 1.0 eq.) was given to ethylene glycol dimethyl ether (20 mL) under inert gas conditions. To the resulting solution, 4,4'-dicarboxyl-2,2'-bipyridine (0.18 g, 0.74 mmol, 1.0 eq.) was added and the reaction mixture was stirred overnight under inert gas atmosphere at 90 °C. After cooling, the solvent was removed *in vacuo* and the resulting solid was dissolved in minimal amounts of ethylene glycol dimethyl ether and given to hexane (100 mL) at 0 °C. The precipitate was isolated *via* centrifugation and dried *in vacuo* to give the orange product of which characterisations matched literature reports.[11]

^{1}H NMR (400 MHz, 300 K, CD_3CN): δ (ppm) = 9.20 (d, ^{3}J = 5.7 Hz, 2H), 8.95 (s, 2H), 8.06 (d, ^{3}J = 5.7 Hz, 2H)

ATR-IR (Re(CO)$_3$) = 2021, 1915 and 1870 cm^{-1}

***5,10,15,20-tetrakis*(4-carboxylphenyl)-porphyrin (2)**

The synthesis was adapted from a literature known procedure.[12] The first step is the synthesis of *tetrakis*(4-methoxycarbonylphenyl)-porphyrin (TPPCOOMe) by adding

pyrrole (3.09 mL, 44.9 mmol, 1.1 eq.) and methyl-p-formylbenzoate (6.93 g, 42.3 mmol, 1.0 eq.) to refluxing propionic acid (100 mL). After refluxing for 22 h under continuous stirring, the precipitate is filtrated and washed with MeOH (30 mL), EtOAc (10 mL) and THF (10 mL) to yield the purple product (5.45 g, 6.44 mmol, 15% yield). Analytics matched literature reports.

^{1}H NMR (400 MHz, $CDCl_3$): δ [ppm] = 8.82 (s, 8H, *β*-pyrrole), 8.45 (d, ^3J = 8.1 Hz, 8H, phenyl), 8.38 (d, ^3J = 8.2 Hz, 8H, phenyl), 4.12 (s, 12H, COOMe), -2.81 (s, 2H, N*H*). Anal. calcd. (%) for TPPCOOMe: C, 73.75; H, 4.52; N, 6.62. Found (%): C, 71.88; H, 4.71; N, 6.06.

In the second step, TPPCOOMe (1.00 g, 1.19 mmol, 1.0 eq.) was dissolved in a 1:1 mixture of THF/MeOH (70 mL). A solution of KOH (3.5 g, 62.4 mmol, 52 eq.) in H_2O (30 mL) was added. The resulting mixture is refluxed for 15 h. After removing the organic solvents in vacuo, the solid is redissolved in H_2O (150 mL) at 90 °C for 15 min. The solution was filtered and acidified with 1 M HCl (100 mL). The resulting green precipitate is filtered and dried to obtain the product, (864 mg, 1.09 mmol, 92% yield). Analytics matched literature reports.

^{1}H NMR (400 MHz, DMSO-*d*$_6$): δ [ppm] = 13.29 (s, 4H, COO*H*), 8.87 (s, 8H, *β*-pyrrole), 8.40 (d, ^3J = 8.3 Hz, 8H, phenyl), 8.36 (d,^3J = 8.4 Hz, 8H, phenyl), –2.94 (s, 2H, N*H*). Anal. calcd. (%) for TCPP: C, 72.91; H, 3.82;

N, 7.09. Found (%): C, 66.76; H, 3.88; N, 6.28. Crystal water: 2 H_2O.

***5,10,15,20-tetrakis*(4-carboxylphenyl)-porphyrin-Zn(II) (2-Zn)**

TPPCOOMe (854 mg, 1.01 mmol, 1.0 eq.) and $ZnCl_2$ (1.75 g, 12.8 mmol, 12.8 eq.) were refluxed in DMF (100 mL) for 6 h. After the mixture was cooled to room temperature, H_2O (150 mL) was added. The dark purple precipitate was filtered and washed with H_2O (2 × 50 mL). The obtained solid was dissolved in $CHCl_3$ (500 mL) and washed with H_2O (3 × 250 mL). The organic layer was dried over anhydrous Na_2SO_4. Subsequently, the solvent was removed by rotary evaporation to yield TPPCOOMe-Zn(II) (276 mg, 303 µmol, 30% yield).

^{1}H NMR (400 MHz, DMSO-*d*$_6$): δ [ppm] = 8.79 (s, 8H, *β* -pyrrole), 8.39 (d, ^3J = 8.2 Hz, 8H, phenyl), 8.33 (d, ^3J = 8.2 Hz, 8H, phenyl), 4.04 (s, 12H, COO*Me*).

Anal. calcd. (%) for TPPCOOMe-Zn: C, 68.61; H, 3.99; N, 6.16. Found (%): C, 65.43; H, 4.15; N, 5.68.

TPPCOOMe-Zn(II) (214 mg, 235 µmol, 1.0 eq.) was dissolved in THF/MeOH 1:1 (15 mL). KOH (691 mg, 12.3 mmol, 52 eq.) was dissolved in H_2O (6 mL) and added to the prior solution. The reaction mixture was refluxed for 5 h under continuous stirring. After the mixture was cooled to room temperature, the organic solvents were removed by rotary evaporation. The crude product was dissolved in H_2O (32 mL) and heated at 90 °C for 10 min. After cooling to room temperature, the aqueous mixture was acidified with 1 M HCl solution (21 mL) and 37% HCl (1 mL) in respective order. The resulting dark green precipitate was isolated by centrifugation and washed with H_2O (8 × 30

mL). The dark green product was dried *in vacuo* overnight (137 mg, 161 μmol, 68% yield).

^{1}H NMR (400 MHz, DMSO-d_6): δ [ppm] = 13.25 (s, 4H, COO*H*), 8.87 (s, 8H, *β*-pyrrole), 8.40 (d, ^3J = 8.2 Hz, 8H, phenyl), 8.36 (d, ^3J = 8.2 Hz, 8H, phenyl).

Anal. calcd. (%) for **2-Zn**: C, 67.50; H, 3.30; N, 6.56. Found (%): C, 65.64; H, 4.01; N, 5.91. Crystal water: 1.65 H_2O.

PCN-222

The synthesis was adapted from a literature known procedure.[13,14] In a 20 mL screw cap vial, **2** (12.5 mg, 0.016 mmol, 1.00 eq.) and $ZrOCl_2$·8 H_2O (23.5 mg, 0.073 mmol, 4.56 eq.) were dissolved in DEF (3 mL). After addition of 4-tert-butylbenzoic acid (1350 mg, 7.57 mmol, 473 eq.), the mixture is ultrasonicated for 10 min. The mixture is heated at 120 °C for 12 h in an oven. The solid is separated by centrifugation and soaked with DMF (3 × 6 mL) and dried in vacuo. The solid (12.2 mg) is dispersed in DMF (8.1 mL) and acidified with 8 M HCl (0.3 mL). The mixture is heated in an oven at 120 °C for 12 h. The purple solid is collected by centrifugation and soaked in DMF (3 × 6 mL) and Acetone (3 × 6 mL) and dried in vacuo to yield the product. Analytics matched literature reports.

PCN-224

The synthesis was adapted from a literature known procedure.[13,14] In a 20 mL screw cap vial, **2** (16.0 mg, 0.020 mmol, 1.00 eq.) and $ZrOCl_2$·8 H_2O (30.0 mg, 0.093 mmol, 4.65 eq.) were dissolved in DMF (4 mL). After addition of formic acid (0.69 mL, 17.2 mmol, 860 eq.) and 3,3-dimethylbutanoic acid (0.81 mL, 6.36 mmol, 318 eq.), the mixture is ultrasonicated for 10 min. The mixture is heated at 120 °C for 12 h in an oven. The solid is separated by centrifugation and soaked with DMF (3 × 6 mL) and dried in vacuo. The solid (31.1mg) is dispersed in DMF (21 mL) and acidified with 8 M HCl (1.6 mL). The mixture is heated in an oven at 120 °C for 12 h. The purple solid is collected by centrifugation and soaked in DMF (3 × 10 mL) and Acetone (3 × 10 mL) and dried in vacuo to yield the product. Analytics matched literature reports.

Metalation of 222 or 224 with Zn

The synthesis was adapted from a literature known procedure.[15] $ZnCl_2$ (59.9 mg for **222** and 22.9 mg for **224**) was dissolved in DMF (3 mL). After addition of the MOF powder (30 mg), the suspension was heated in an oven at 100°C for 24 °C. The product was isolated by centrifugation and washed with DMF (3 × 6 mL) and acetone (3 × 6 mL). Drying in vacuo yielded the product.

1,3-Dimethyl-2-phenyl-2,3-dihydro-*1H*-benzo[*d*]imidazole (BIH)

Polyphosphoric acid (10.5 g), benzoic acid (3.07 g, 25.1 mmol, 1.0 eq.) and *o*-phenylenediamine (2.71 g, 25.1 mmol, 1.0 eq.) were stirred at 180 °C for 2 h. Subsequently, ammonia solution (50 mL, 6% in H_2O) was added, and the purple product was isolated and washed with ammonia solution and dried *in vacuo* to yield 2-

phenyl-*1H*-benzo[*d*]imidazole used without further purification (4.83 g, 24.8 mmol, 99% yield).

NaOH (1.04 g, 25.8 mmol, 1.04 eq.) and methanol (25 mL) were added to 2-phenyl-1H-benzo[d]imidazole (4.83 g, 24.8 mmol, 1.0 eq.). Methyl iodide (5.64 mL, 90.8 mmol, 3.65 eq.) was added to the suspension and refluxed for 48 h. The solvent was removed *in vacuo*, the crude product was dissolved in hot ethanol/H_2O (5/1 v/v, 160 mL) and activated carbon was added. After 30 min, the slurry was filtered hot over a Celite® pad, the solvent was removed *in vacuo* and the resulting solid was recrystallized from ethanol to yield 1,3-dimethyl-2-phenyl-*1H*-benzo[*d*]imidazole-3-ium iodide (5.44 g, 15.5 mmol, 62% yield) as light-yellow needles.

^{1}H NMR (400 MHz, 300 K, DMSO-d$_6$) δ (ppm) = 8.13 (m, 2H), 7.76 (m, 2H), 7.92 (m, 5H), 3.92 (s, 6H)

1,3-dimethyl-2-phenyl-*1H*-benzo[*d*]imidazole-3-ium iodide (5.44 g, 15.5 mmol, 1.0 eq.) was dissolved in dry methanol (150 mL) and sodium borohydride (2.16 g, 57.0 mmol, 3.7 eq.) was added under inert conditions. After 1 h of stirring at room temperature, the solvent was removed *in vacuo* and the crude product recrystallized three times from ethanol/H_2O (2/1 v/v) to yield 1,3-dimethyl-2-phenyl-2,3-dihydro-*1H*-benzo[*d*]imidazole (3.1 g, 13.8 mmol, 89%) as white needles.

^{1}H NMR (400 MHz, 300 K, DMSO-d$_6$): δ (ppm) = 7.55 (m, 2H), 7.44 (m, 3H), 6.61 (m, 2H), 6.45 (m, 2H), 4.87 (s, 1H), 2.48 (s, 6H)

Elemental analysis: Calculated: C 80.32; H 7.19; N 12.49. Found: C 79.93; H 7.20; N 12.41.

$K_3[Fe(C_2O_4)_3] \cdot 3\ H_2O$

The synthesis was adapted from literature procedures.[2,3] A 1.5 M potassium oxalate (27.6 g $K_2C_2O_4 \cdot H_2O$ in 100 mL deionised H_2O) and a 1.5 M $FeCl_3$ (24.3 g $FeCl_3$ in 100 mL deionised H_2O) solution were prepared. The two solutions were then combined under stirring in a 3/1 volumetric ratio (potassium oxalate/ferric chloride) in the dark. After 2 hours of stirring at room temperature the resulting green precipitate was filtered off and recrystallised three times from warm water. The solid was dried at 40 °C and stored in the dark.

Elemental analysis: Calculated: C 14.67; H 1.23. Found: C 14.77; H 1.11.

$Zr_6(OH)_4O_4(OMc)_{12}$ (isolated Zr_6-oxo-node, OMc = methacrylate)

The synthesis followed a literature procedure.[16] Accordingly, Zr(IV) propoxide (1 mL, 70% w/v in *n*-propanol, 3.1 mmol) and methacrylic acid (1 mL 11.8 mmol, 5.3 eq.) were mixed in a flask under *Schlenk* conditions. After two weeks without stirring at room temperature the formed crystals were collected by filtration and washed with small quantities of *n*-propanol. After drying in vacuo, 860 mg $Zr_6O_4(OH)_4(OMc)_{12}$ (0.51 mmol, 98 %) was received. Analytics matched literature reports.[16,17]

Molecular catalyst loading in 222-Zn or 224-Zn

A 0.1 mM solution of **1** in MeCN (15 mL) was added to powder samples of the respective activated MOF sample (10.0 mg) in VWR® TraceClean® 25 mL vials. After

24 h in the dark, the suspension was centrifuged, and the supernatant removed. The resulting powder was washed with fresh MeCN (3 × 7 mL) for 2 h per wash cycle and then dried overnight in vacuo.

For control experiments with *fac*-ReBr$(CO)_3$(4,4'-dicarboxy-2,2'-bipyridine) this procedure was analogously used with 10 mL of a 0.1 mM solution.

3. Supporting Figures

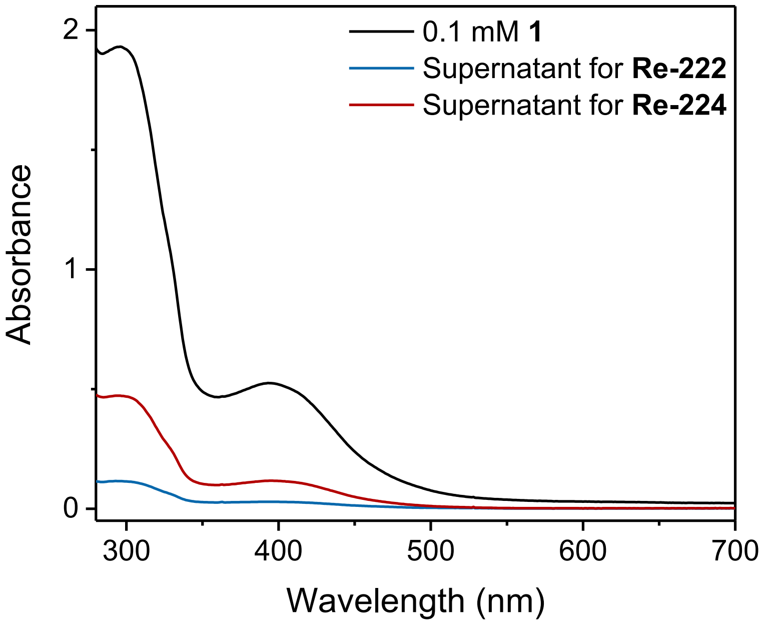

Figure S1: UV-vis spectra of a MeCN solution (15 mL) containing **1** (0.1 mM) with **222-Zn** or **224-Zn** (10.0 mg) before and after the anchoring procedure (24 h soaking).

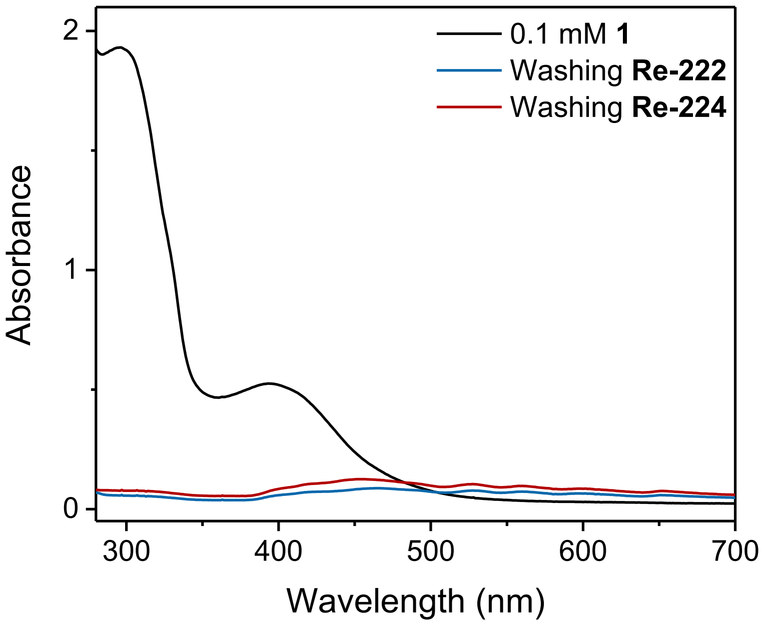

Figure S2: UV-vis spectra after washing **Re-222** and **Re-224** with MeCN (7 mL) to test for leaching and verify stable dyadic anchoring.

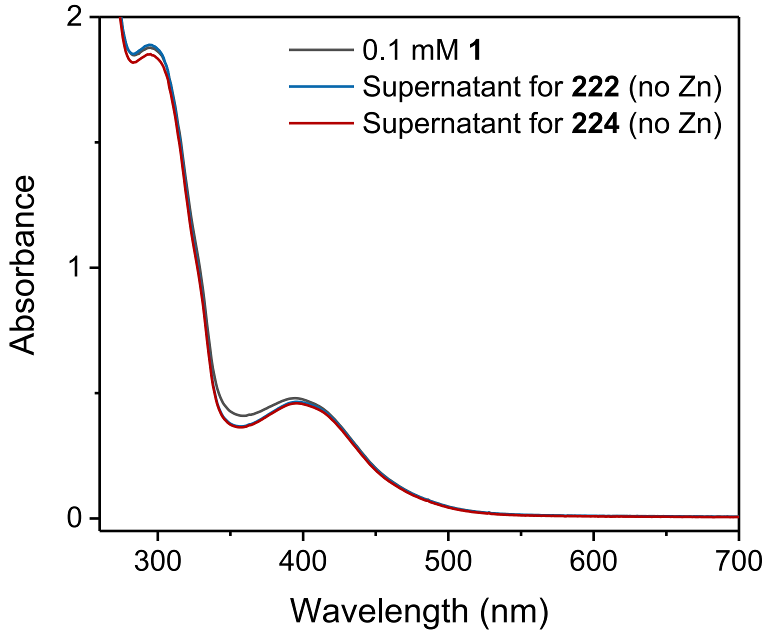

Figure S3: UV-vis spectra of a MeCN solution (15 mL) containing **1** (0.1 mM) with **222** or **224** (10.0 mg) before and after soaking the powder for 24 h. Control experiment verifying the dyadic binding of **1** to Zn.

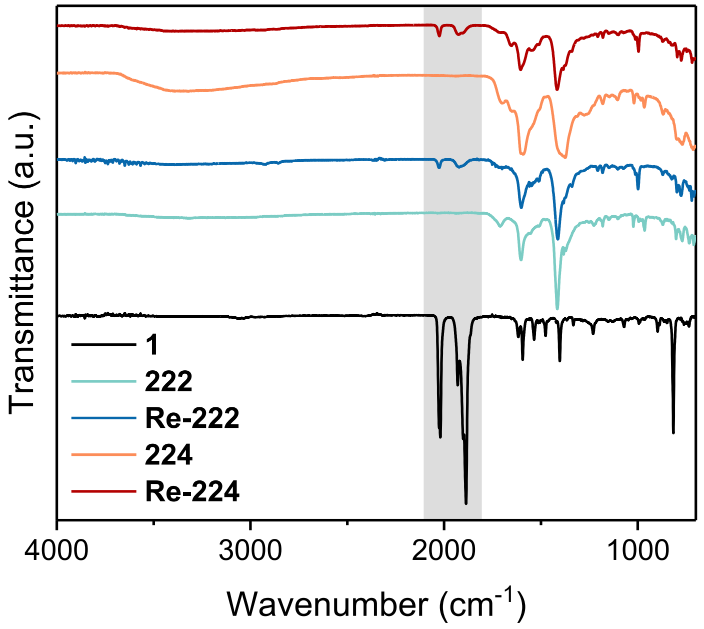

Figure S4: Full ATR-IR spectra of pristine and loaded PCN-222 and -224 hybrids. Including the reference for the pure catalyst **1** (black) with the characteristic region for $Re(CO)_3$ bands highlighted in grey.

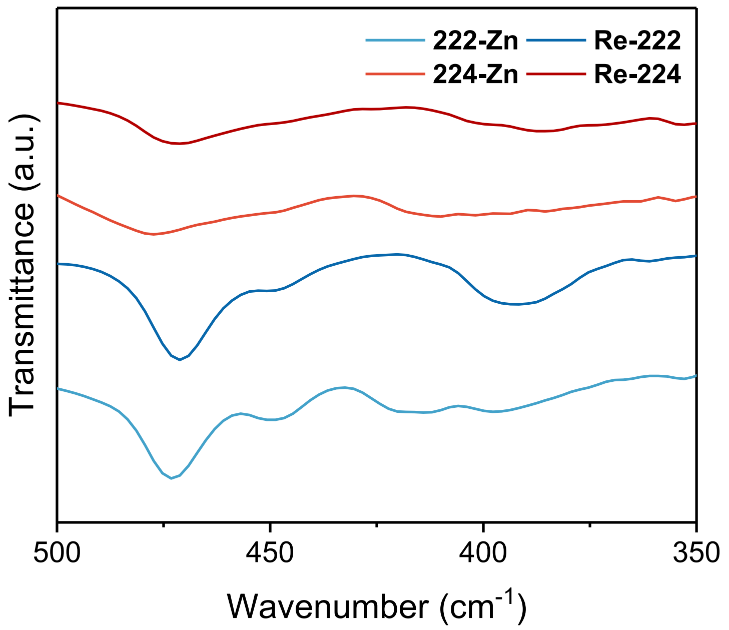

Figure S5: ATR-IR spectra of **222-Zn**, **Re-222**, **224-Zn**, and **Re-224** in the Zn-N region between ~350-400 cm^{-1}.[18] 222-based: Peak shift from 398 cm^{-1} (**222-Zn**) to 394 cm^{-1} (**Re-222**). 224-based: Peak shift from 395 cm^{-1} (**224-Zn**) to 390 cm^{-1} (**Re-224**). These showcase a shift in bands after Re catalyst loading, evidencing significant coordination between TCPP-Zn and the catalyst, forming the dyad.

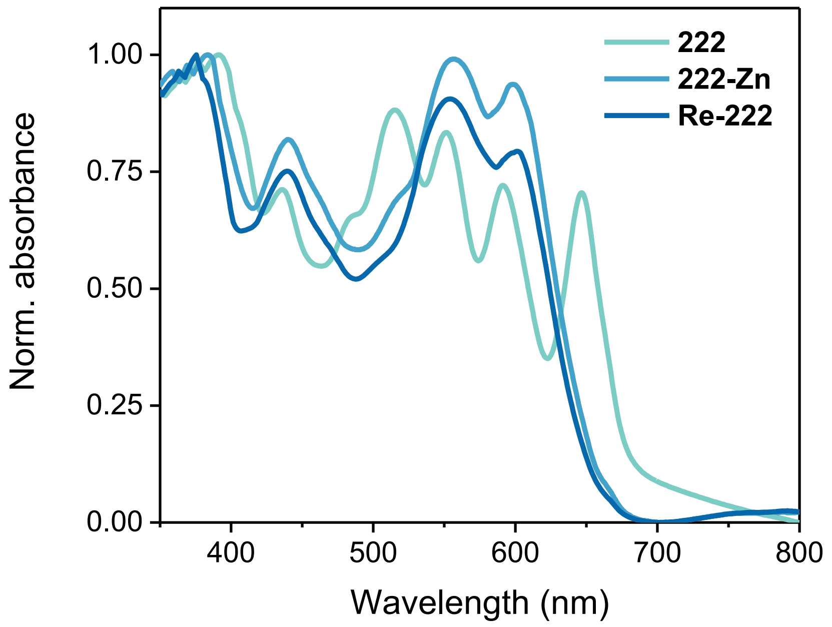

Figure S6: Solid-state UV-vis spectra of **222**, **222-Zn** and **Re-222**.

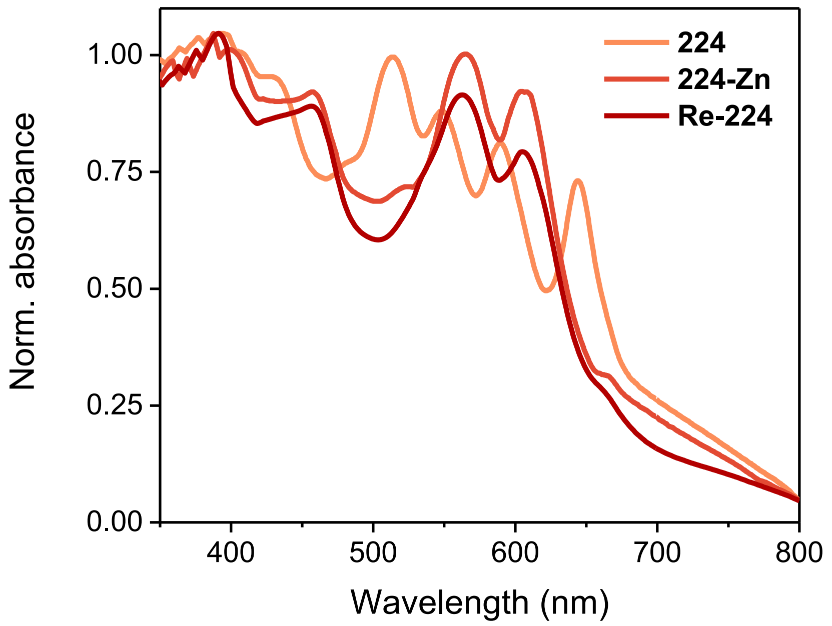

Figure S7: Solid-state UV-vis spectra of **224**, **224-Zn** and **Re-224**.

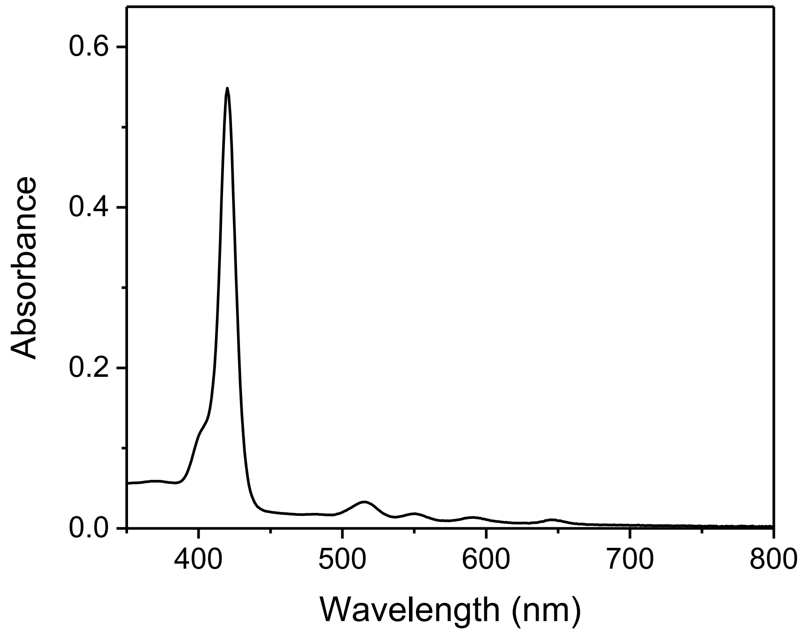

Figure S8: UV-vis spectrum of pure **2** in THF.

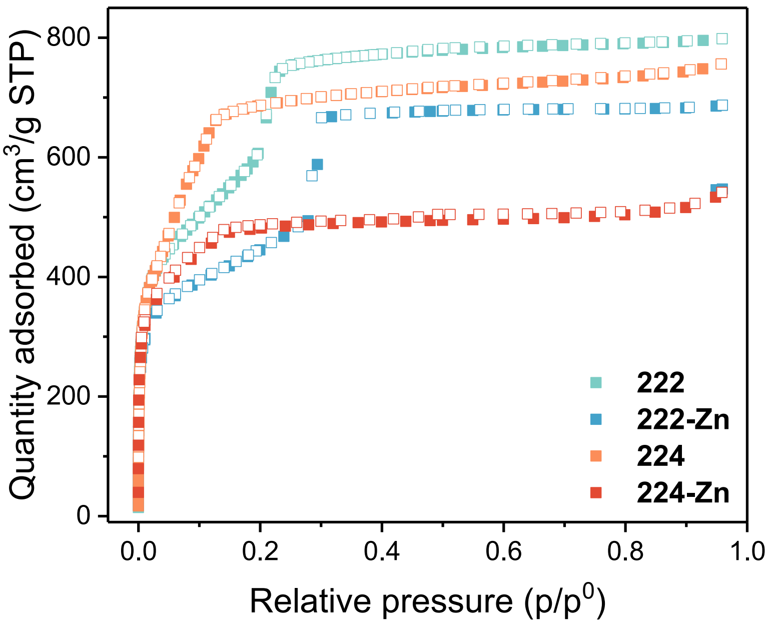

Figure S9: N_2 adsorption isotherms at 77 K for pristine **222**, **222-Zn**, **224** and **224-Zn** after activation at 120 °C in vacuo for 12 hours. The BET surface areas are calculated as 1989.4 ± 7.7 $m^2 \cdot g^{-1}$ (**222**), 1553.6 ± 3.3 $m^2 \cdot g^{-1}$ (**222-Zn**), 1978.0 ± 25.9 $m^2 \cdot g^{-1}$ (**224**), 1752.1 ± 15.7 $m^2 \cdot g^{-1}$ (**224-Zn**).

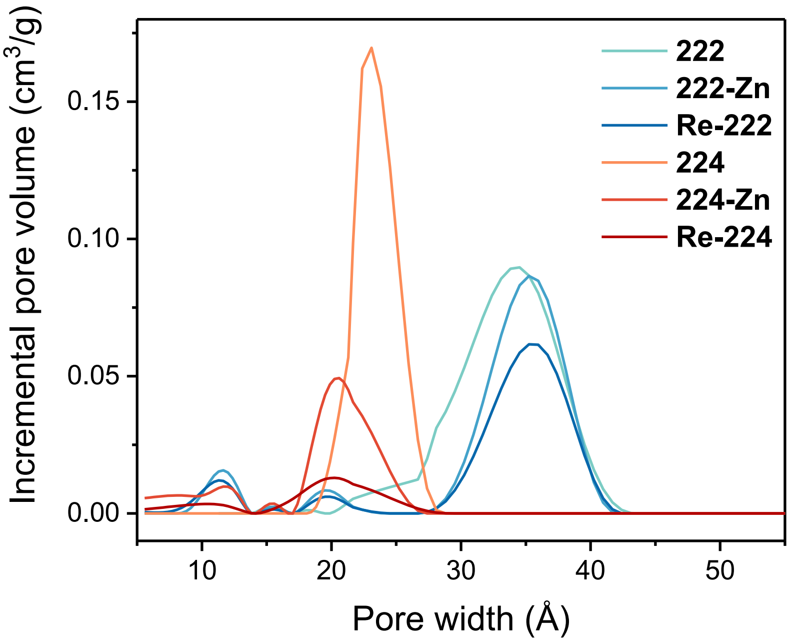

Figure S10: Calculated pore size distributions from N_2 adsorption isotherms using sets of theoretical isotherms (kernel) derived from 2D-NLDFT-based methods for specific pore sizes and geometry. As an approximation, cylindrical pores on an oxide surface were assumed for all materials to allow comparability within this series.

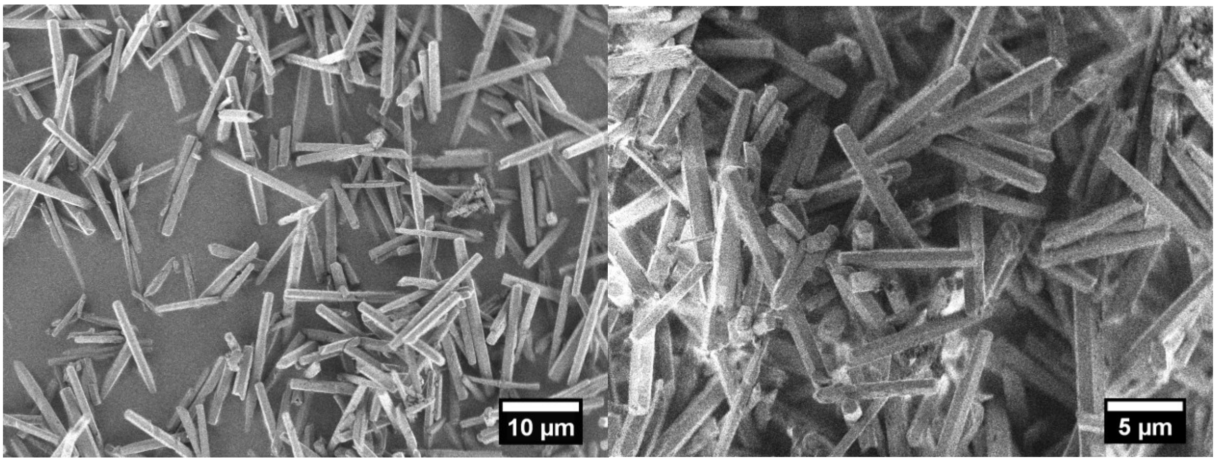

Figure S11: SEM images of **222** and different magnifications.

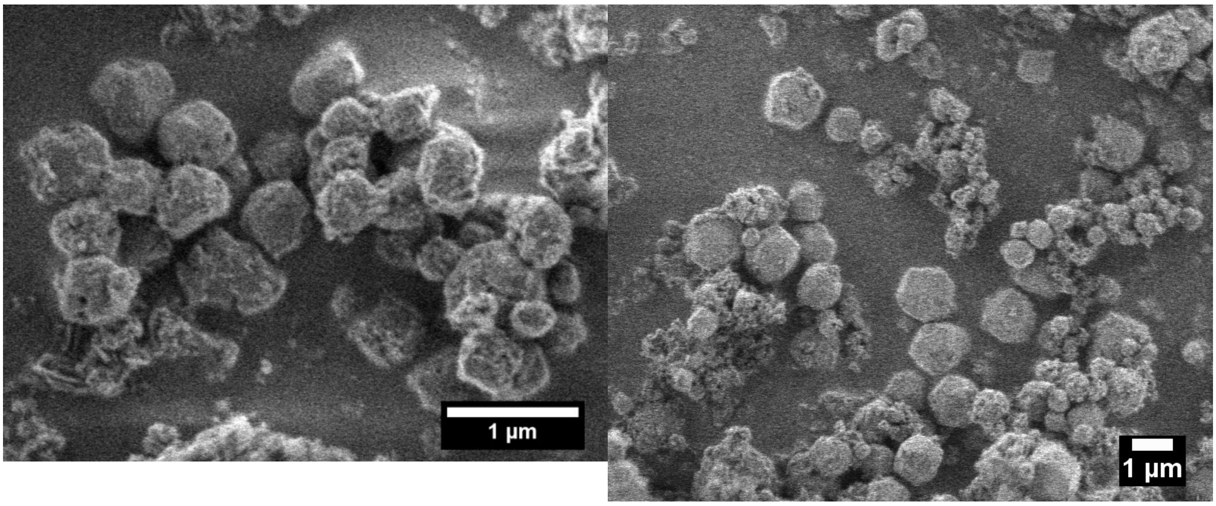

Figure S12: SEM images of **224** and different magnifications.

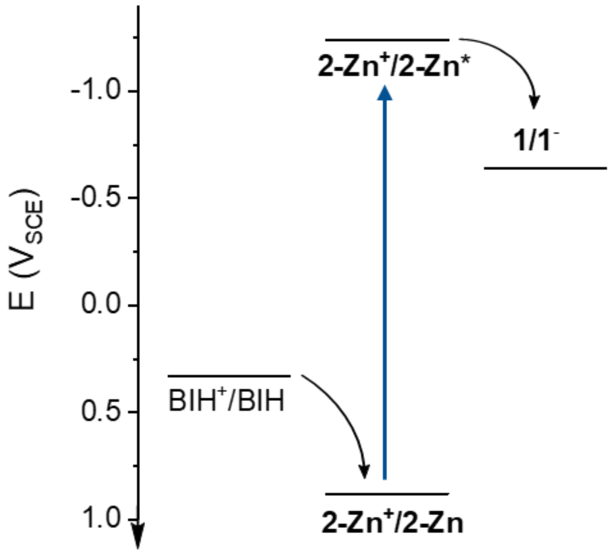

Figure S13: Potential representation of BIH (SED), **2-Zn**, and **1** showing thermodynamic feasibility of directional electron transfer.[10,19] Note that for **2-Zn*** the S_1 state is shown and the blue arrow represents photoexcitation.

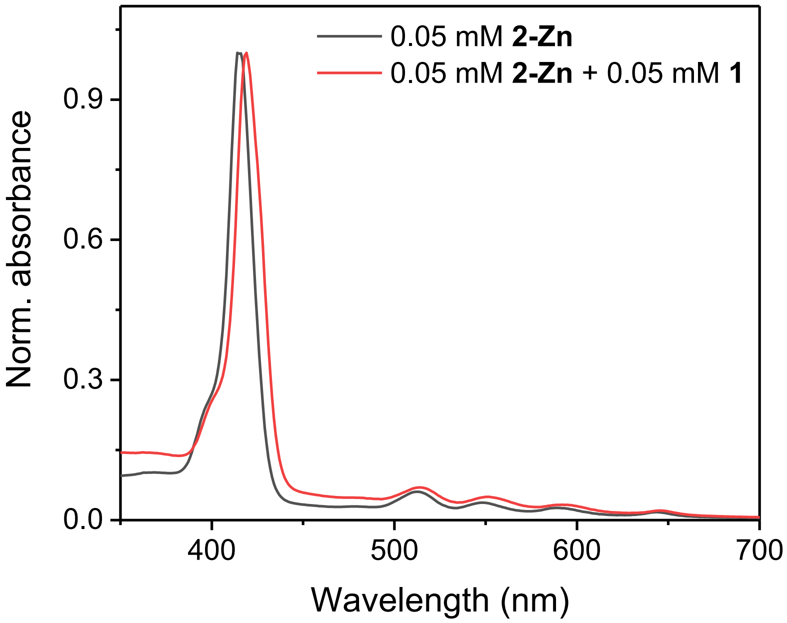

Figure S14: UV-vis spectra of pure **2-Zn** (0.05 mM, black) and equimolar quantities of **1** and **2-Zn** (red) in MeCN/DMF (40/1 v/v). Absorbance normalised on the Soret band.

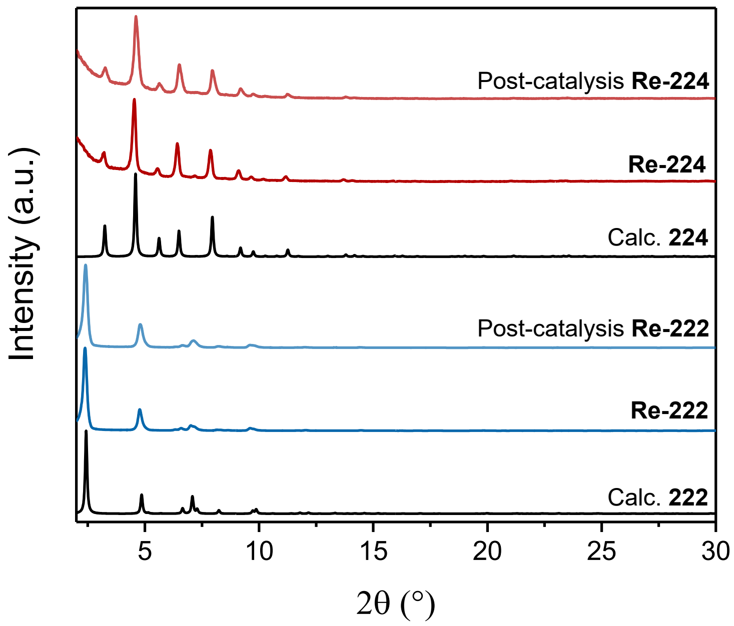

Figure S15: Pre- and post-catalysis PXRD patterns for **Re-222** and **Re-224**. Standard catalysis conditions for post-catalysis samples: 4 mL MeCN, 0.12 mL H_2O, 1 mmol BIH, 430-740 nm irradiation, 72 h.

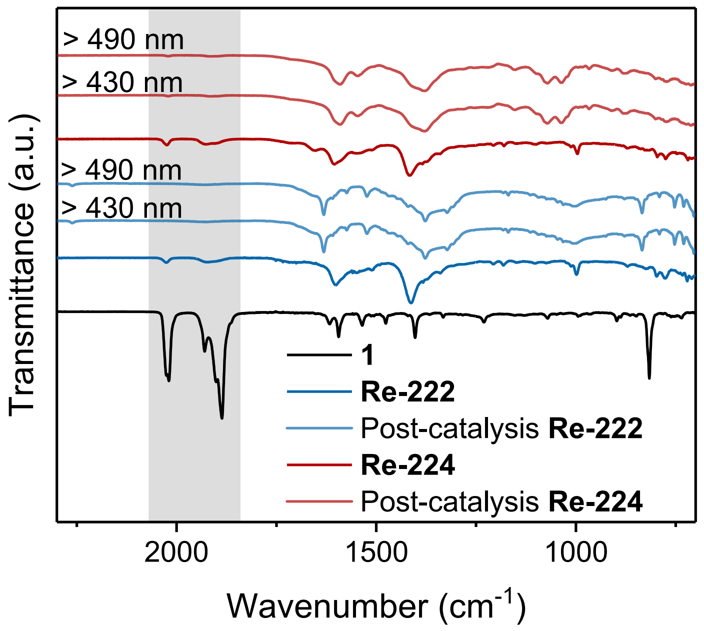

Figure S16: Pre- and post-catalysis ATR-IR spectra for **Re-222** and **Re-224**. Standard catalysis conditions for post-catalysis samples: 4 mL MeCN, 0.12 mL H_2O, 1 mmol BIH, irradiation as specified, 72 h.

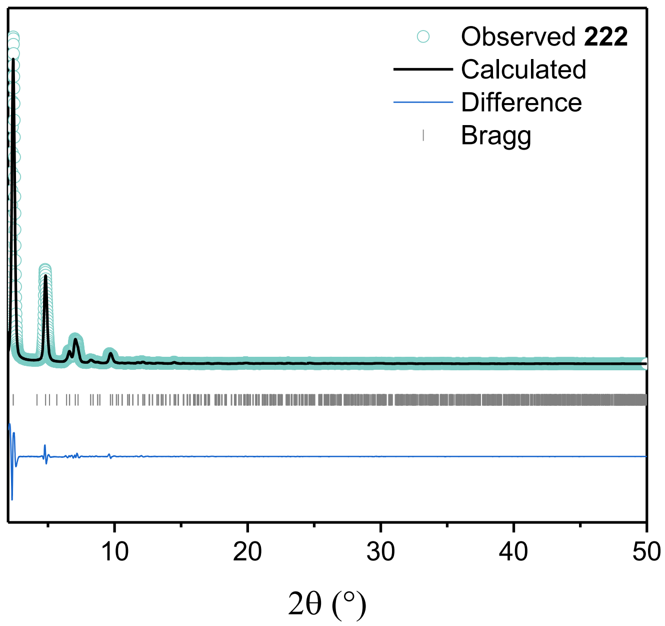

Figure S17: Le Bail refinement for pristine **222**. Performed with *JANA2006* from measured PXRD data. Goodness of fit (GOF) = 0.255, R_p = 5.4%, R_{wp} = 8.3%.

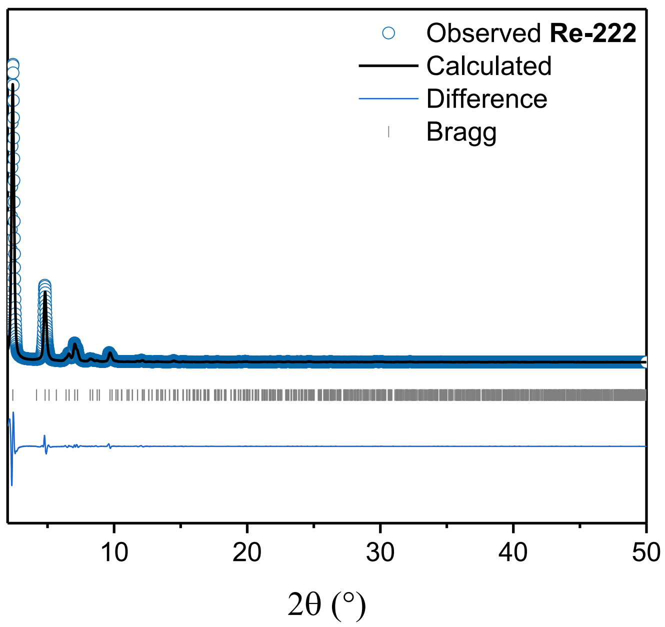

Figure S18: Le Bail refinement for **Re-222**. Performed with *JANA2006* from measured PXRD data. Goodness of fit (GOF) = 0.269, R_p = 6.0%, R_{wp} = 8.6%.

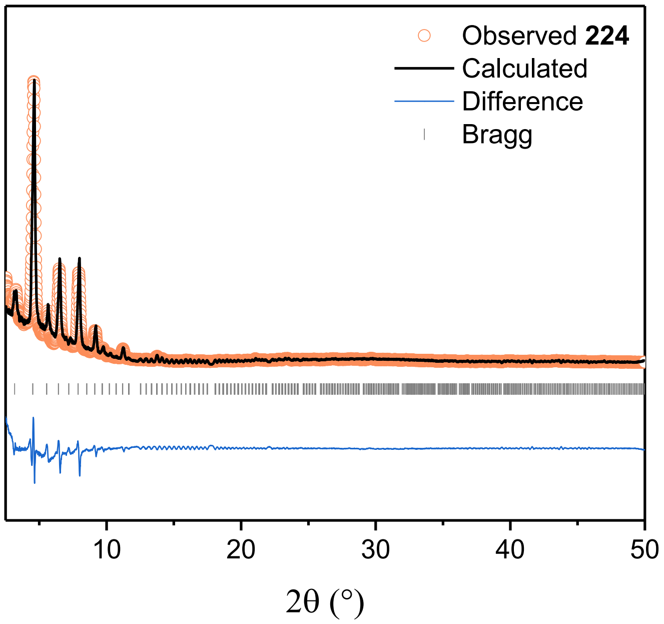

Figure S19: Le Bail refinement for pristine **224**. Performed with *JANA2006* from measured PXRD data. Deviations in difference curve towards lower angles caused by air scattering. Goodness of fit (GOF) = 0.230, R_p = 7.0%, R_{wp} = 9.7%.

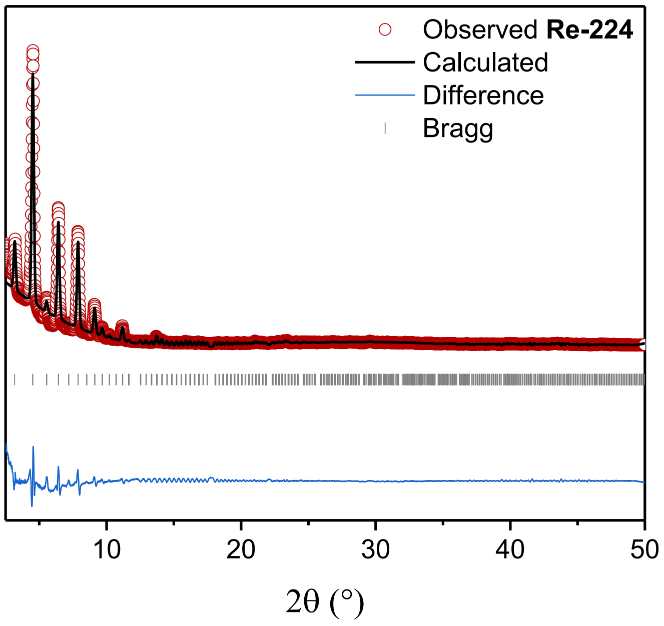

Figure S20: Le Bail refinement for **Re-224**. Performed with *JANA2006* from measured PXRD data. Deviations in difference curve towards lower angles caused by air scattering. Goodness of fit (GOF) = 0.205, R_p = 7.1%, R_{wp} = 9.8%.

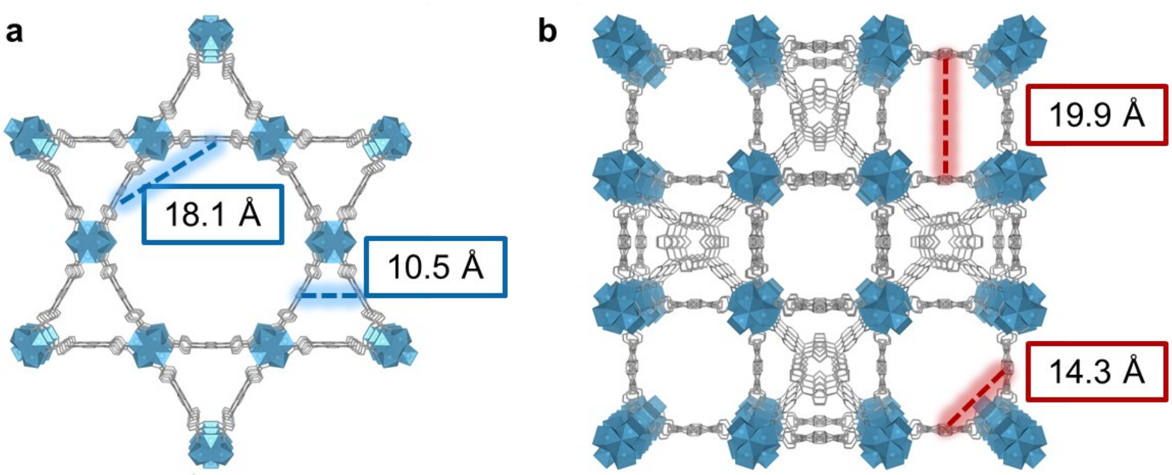

Figure S21: Additional distances between porphyrin chromophores in **222** (**a**) and **224** (**b**) not related to translational exciton hopping along the crystallographic *c*-axis.

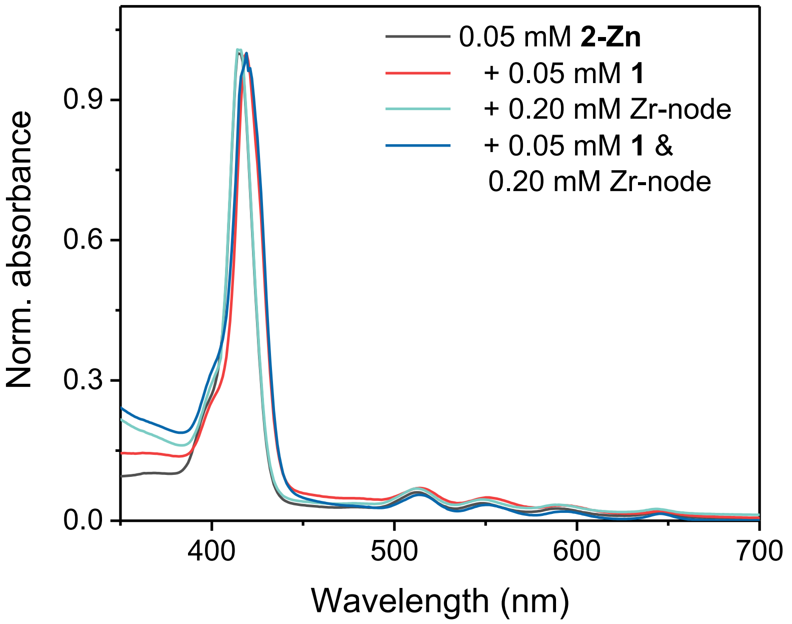

Figure S22: UV-Vis spectra of MeCN/DMF (40/1 v/v) solutions containing **2-Zn** (0.05 mM); **2-Zn** (0.05 mM) + **1** (0.05 mM); **2-Zn** (0.05 mM) + the Zr_6-oxo-node (0.2 mM); and **2-Zn** (0.05 mM) + **1** (0.05 mM) + the Zr_6-oxo-node (0.2 mM). Conditions chosen to be identical to luminescence measurements. Absorbance normalised on the Soret band to show no effect on porphyrin absorption features.

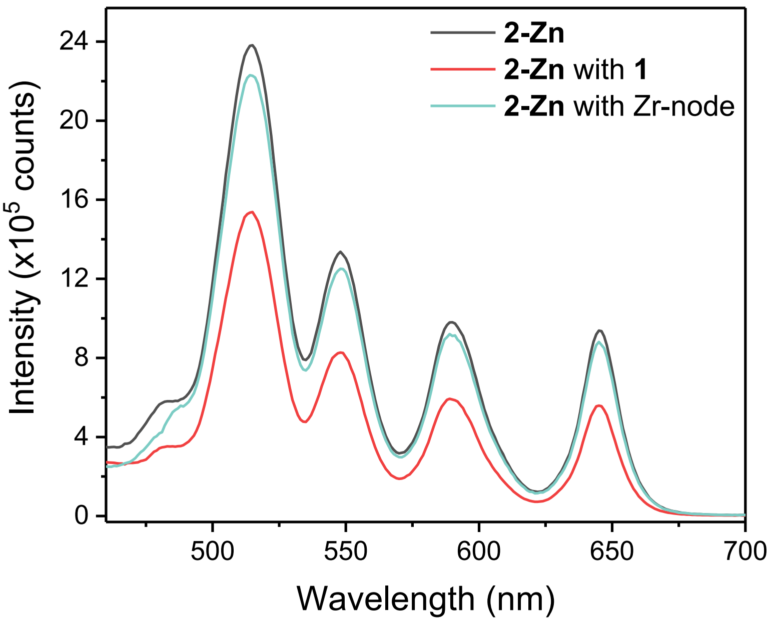

Figure S23: Action fluorescence spectra (emission detector λ = 720 nm) of MeCN/DMF (40/1 v/v) solutions containing **2-Zn** (0.05 mM); **2-Zn** (0.05 mM) + **1** (0.05 mM); and **2-Zn** (0.05 mM) + the Zr_6-oxo-node (0.2 mM).

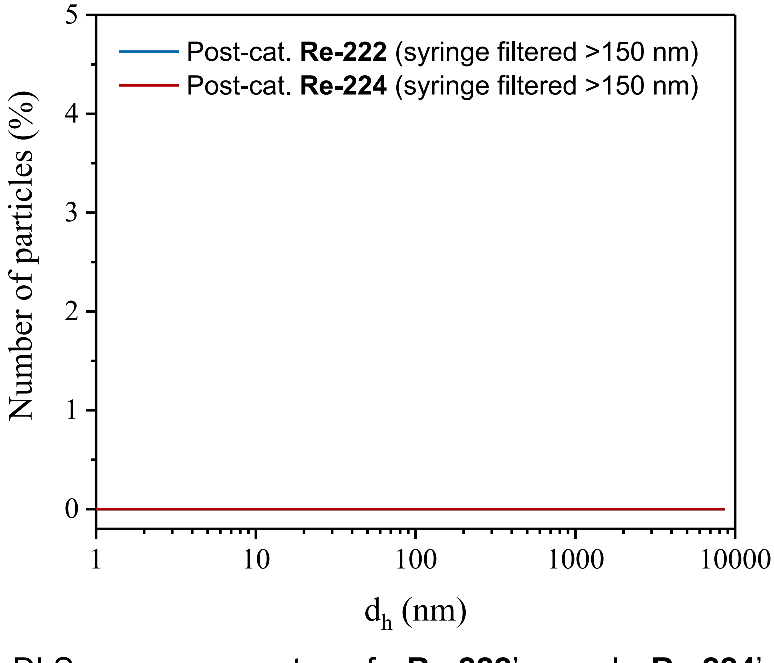

Figure S24: DLS measurements of **Re-222**'s and **Re-224**'s post-catalysis supernatants. Measurement performed after syringe-filtering the solution to remove particles >150 nm to increase resolution for small nanoparticles. Standard catalysis conditions: 4 mL MeCN, 0.12 mL H_2O, 1 mmol BIH, > 490 nm, 80 h.

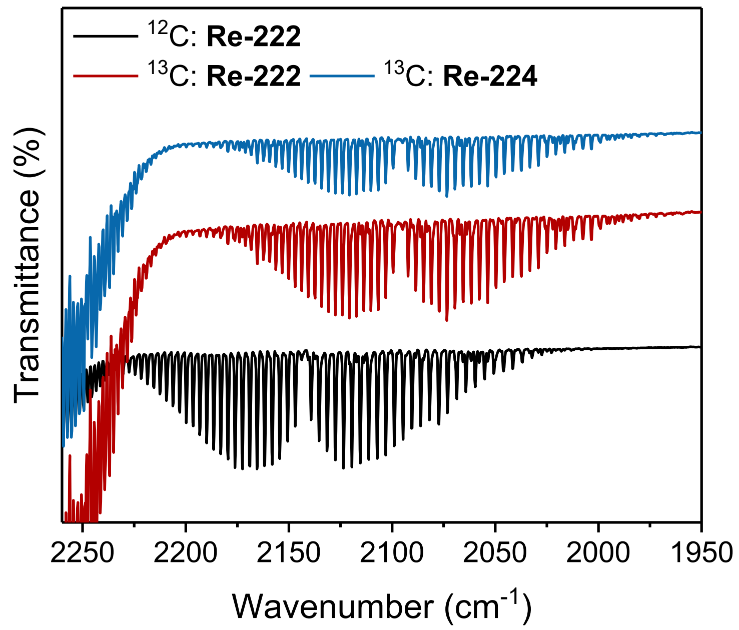

Figure S25: Reaction headspace gas-phase IR spectra after 24 h of catalysis with $^{12}CO_2$ or labelled $^{13}CO_2$. Standard catalysis conditions: 4 mL MeCN, 0.12 mL H_2O, 1 mmol BIH, 490-740 nm irradiation.

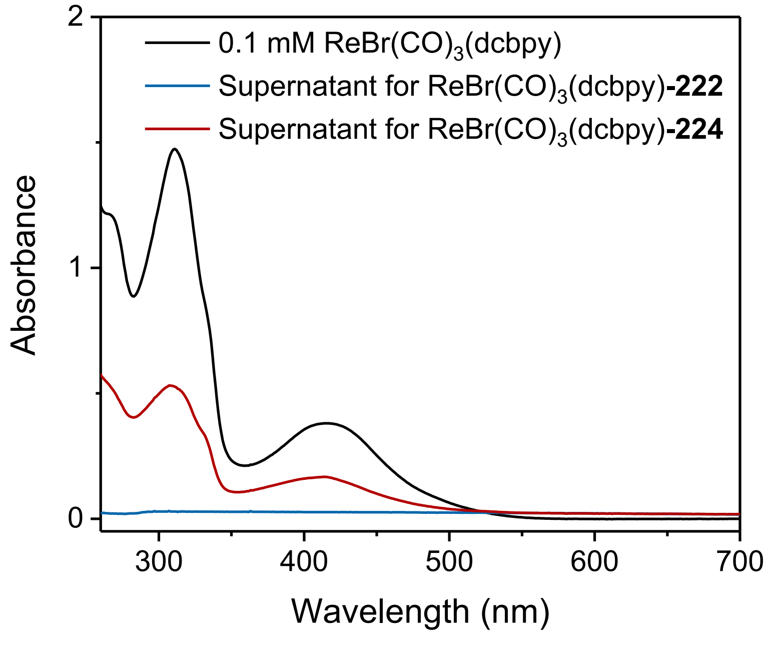

Figure S26: UV-Vis spectra of a 0.1 mM MeCN solution with $ReBr(CO)_3$(dcbpy) and after 24 h upon adding **222** (10.0 mg) or **224** (10.0 mg), respectively.

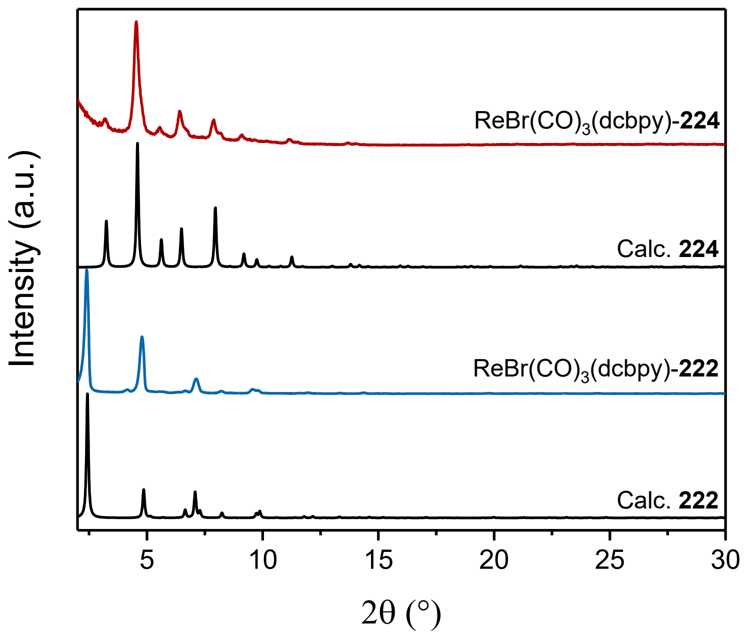

Figure S27: PXRD patterns of MOF assemblies loaded with $ReBr(CO)_3(dcbpy)$ and calculated patterns for pristine **222** and **224**.

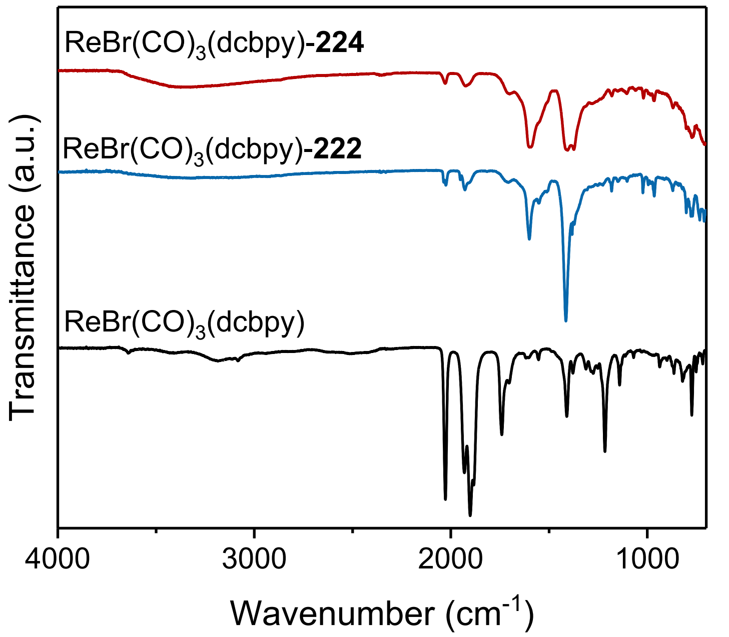

Figure S28: ATR-IR spectra of MOF assemblies loaded with $ReBr(CO)_3(dcbpy)$ and pure $ReBr(CO)_3(dcbpy)$.

4. Supporting Tables

Table S1: ICP-MS measurement values for PCN MOFs and MOF assemblies.

Sample	Zn mass (µg)	Re mass (µg)	Sample mass (mg)	Zn (nmol· mg_{MOF}^{-1})	Zr/ Zn	Re (nmol· mg_{MOF}^{-1})	Zn/ Re	Catalyst/ Linker[a]
Pristine PCN-222	n.-d.	n.-d.	1.07	/		/	/	/
**222-Zn**	57.34 ± 0.73	n.-d.	0.93	943.0 ± 12.0	1.8	/	/	/
**Re-222**	65.75 ± 0.78	15.80 ± 0.19	1.04	966.9 ± 11.5		81.57 ± 0.98	11.9	0.103
Pristine PCN-224	n.-d.	n.-d.	1.11	/		/	/	/
**224-Zn**	93.11 ± 0.97	n.-d.	1.46	975.4 ± 10.2	1.9	/	/	/
**Re-224**	55.94 ± 0.68	17.54 ± 0.22	1.25	684.5 ± 8.3		75.37 ± 0.95	9.1	0.109
$ReBr(CO)_3$(dcbpy) **-222**	/	7.04 ± 0.09	1.08	/		35.02 ± 0.45	/	/
$ReBr(CO)_3$(dcbpy) **-224**	/	4.28 ± 0.09	1.22	/		18.82 ± 0.17	/	/

[a]*Catalyst/Linker ratios are calculated from the ideal sum formula of PCN-222 and PCN-224 and are only for estimation since defects are not considered.*

Formulas applied for calculations with exemplary data:

$$n(\mathrm{Re}_{\text{per mg of MOF}}) = \frac{m_{Re}}{M_{Re}} \cdot \frac{1}{m_{Sample}} = \frac{15.80\ \mu\mathrm{g}}{186.21\,\frac{\mathrm{g}}{\mathrm{mol}}} \cdot \frac{1}{1.04\ \mathrm{mg_{MOF}}} = 81.57\,\frac{\mathrm{nmol}}{\mathrm{mg_{MOF}}}$$

Table S2: Performance data for homogeneous photocatalytic reduction of CO_2. Standard conditions, unless specified otherwise: 0.1 mM **1** in MeCN and 0.1 mM **2-Zn** in MeCN (4 mL total volume), 0.12 mL deionised H_2O, BIH (1 mmol, 225 mg). The respective errors are calculated from averaging three repetitions under identical conditions. TONs based on mol Re.

Sample	λ (nm)	Comment	TON_{CO}	Formate
**1**	> 430		4.9 ± 0.3	n.-d.
**1** & **2-Zn** (1:1 mol%)	> 430		10.4 ± 0.4	n.-d.
	> 430	No BIH	n.-d.	n.-d.
	> 430	No irradiation	n.-d.	n.-d.
	> 490		10.4 ± 0.3	n.-d.
**Zr_6 | 2-Zn | 1** (4|1|1 mol%)	> 430		0.8 ± 0.1	traces
	> 490		10.4 ± 0.5	n.-d.

Table S3: Performance data for colloidal photocatalytic reduction of CO_2. Standard conditions, unless specified otherwise: 1.5 mg MOF assembly, 4 mL MeCN, 0.12 mL deionised H_2O, BIH (1 mmol, 225 mg). The respective errors are calculated by averaging three repetitions under identical conditions and the catalyst quantity errors from ICP-MS analysis. $TONs_{CO}$ based on mol Re, $TONs_{HCOO^-}$ based on Zr_6-oxo nodes (calculations provided below), and rates averaged over the first 2 h.

Sample	λ (nm)	Comment	CO ($\mu mol \cdot g^{-1} \cdot h^{-1}$)	TON_{CO}	Formate ($\mu mol \cdot g^{-1} \cdot h^{-1}$)	TON_{HCOO^-} (80 h)[a]
**222-Zn**	> 430		n.-d.	n.-d.	26.7 ± 0.6	5.4 ± 0.1
	> 430	No BIH		n.-d.	n.-d.	n.-d.
	> 430	No irrad.		n.-d.	n.-d.	n.-d.
**Re-222**	> 430		4.0 ± 0.7	0.9 ± 0.2	27.0 ± 0.7	5.5 ± 0.1
	> 490		374 ± 26	101 ± 7	0.8 ± 0.4	0.2 ± 0.1
	> 490	No BIH	n.-d.	n.-d.	n.-d.	n.-d.
	> 490	No irrad.	n.-d.	n.-d.	n.-d.	n.-d.
**224-Zn**	> 430		n.-d.	n.-d.	8.3 ± 0.4	1.4 ± 0.1
	> 430	No BIH	n.-d.	n.-d.	n.-d.	n.-d.
	> 430	No irrad.	n.-d.	n.-d.	n.-d.	n.-d.
**Re-224**	> 430		1.5 ± 0.3	0.5 ± 0.1	8.8 ± 0.5	1.5 ± 0.1
	> 490		24.2 ± 2.1	11.5 ± 1.0	0.8 ± 0.2	0.1 ± 0.1
	> 490	No BIH	n.-d.	n.-d.	n.-d.	n.-d.
	> 490	No irrad.	n.-d.	n.-d.	n.-d.	n.-d.
$ReBr(CO)_3$ (dcbpy)-**222**	> 430			2.0 ± 0.3	27.4 ± 0.5	
	> 490			351 ± 24	0.6 ± 0.2	
$ReBr(CO)_3$ (dcbpy)-**224**	> 430			1.0 ± 0.2	8.7 ± 0.4	
	> 490			15.6 ± 0.8	0.2 ± 0.2	

[a]Note that the MOF samples are still active in formate production after 80 h, the TON stated does not reflect the entire lifetime of the catalyst.

Calculation of Turnover Numbers (TONs)

For every photocatalytic experiment the TONs were calculated as follows. For CO, the exact molar catalyst amount was obtained by multiplying the nmol/mg_{MOF} values from ICP-MS analysis with the weigh-in. Subsequently, the GC headspace analysis provided area% results for each gas present. For CO these were then converted to vol% (calibrated with reference gases from *Air Products GmbH*), volume, and moles in turn with the following formulas. To account for solvated CO in MeCN, Henry's law was used with a Henry constant of 2507 bar·mol_{MeCN}·mol_{CO}^{-1}.[7,8] An exemplary TON calculation for CO_2 to CO is provided below for **Re-222** under > 490 nm irradiation with full measurement data in Table S4.

$$n_{\mathrm{Re}}[\mathrm{nmol}] = n_{\mathrm{Re}}\left[\frac{\mathrm{nmol}}{\mathrm{mg_{MOF}}}\right] \cdot m_{\mathrm{Sample}} = 81.57\ \frac{\mathrm{nmol}}{\mathrm{mg_{MOF}}} \cdot 1.50\ \mathrm{mg} = 122\ \mathrm{nmol}$$

$$Vol\%_{\mathrm{CO}} = Area\%_{\mathrm{CO}} \cdot 1.195 = 0.243 \cdot 1.195 = 0.290$$

$$V_{\mathrm{CO,Gas}}[\mathrm{mL}] = \frac{Vol\%_{\mathrm{CO}}}{100} \cdot V_{Headspace} = \frac{0.290}{100} \cdot 70.88\ \mathrm{mL} = 0.206\ \mathrm{mL}$$

$$n_{\mathrm{CO,Gas}}[\mu\mathrm{mol}] = \frac{p \cdot V_{\mathrm{CO}}}{R \cdot T} = \frac{1.45 \cdot 10^{5}\mathrm{Pa} \cdot 0.206\ \mathrm{mL}}{8.314\ \frac{\mathrm{J}}{\mathrm{K \cdot mol}} \cdot 293.15\ \mathrm{K}} = 12.2\ \mu\mathrm{mol}$$

Note: The headspace volume $V_{Headspace}$ is obtained by subtracting the solvent volume (here: 4.12 mL) from the maximum volume the reaction vial holds when filled completely (here 75 mL in a *Schlenk* flask). The pressure p includes the applied CO_2 overpressure for a total pressure of 1.45 bar.

$$p_{\mathrm{CO}}[\mathrm{mbar}] = \frac{n_{\mathrm{CO,Gas}} \cdot R \cdot T}{V_{Headspace}} = \frac{12.2\ \mu\mathrm{mol} \cdot 8.314\ \frac{\mathrm{J}}{\mathrm{K \cdot mol}} \cdot 293.15\ \mathrm{K}}{71.0\ \mathrm{mL}} = 3.0\ \mathrm{mbar}$$

$$\mathrm{c_{CO,Solv}}\left[\mathrm{mol_{CO}} \cdot \mathrm{mol_{MeCN}^{-1}}\right] = \frac{p_{\mathrm{CO}}}{K_{\mathrm{Henry}}} = \frac{3.0\ \mathrm{mbar}}{2507\ \mathrm{bar} \cdot \mathrm{mol_{MeCN}} \cdot \mathrm{mol_{CO}^{-1}}}$$
$$= 1.7\ \mu\mathrm{mol_{CO}} \cdot \mathrm{mol_{MeCN}^{-1}}$$

$$\mathrm{n_{CO,Solv}}[\mu\mathrm{mol}] = \mathrm{c_{CO,Solv}} \cdot n_{\mathrm{MeCN}} = \mathrm{c_{CO,Solv}} \cdot \frac{V_{\mathrm{MeCN}}}{M_{\mathrm{MeCN}}}$$
$$= 1.7\ \mu\mathrm{mol_{CO}} \cdot \mathrm{mol_{MeCN}^{-1}} \cdot \frac{4\ \mathrm{mL}}{41.05\ \mathrm{g} \cdot \mathrm{mol}^{-1}} = 0.2\ \mu\mathrm{mol}$$

$$n_{\mathrm{CO,Total}}[\mu\mathrm{mol}] = n_{\mathrm{CO,Gas}}[\mu\mathrm{mol}] + \mathrm{n_{CO,Solv}}[\mu\mathrm{mol}] = 12.2\ \mu\mathrm{mol} + 0.2\ \mu\mathrm{mol} = 12.4\ \mu\mathrm{mol}$$

$$TON_{\mathrm{CO}} = \frac{n_{\mathrm{CO,Total}}[\mu\mathrm{mol}]}{n_{\mathrm{Re}}[\mu\mathrm{mol}]} = \frac{12.4\ \mu\mathrm{mol}}{0.122\ \mu\mathrm{mol}} = 101$$

For formate, the amount of evolved product (mol) was determined by ^{1}H NMR and divided by the quantity of Zr_6-oxo nodes (mol) present during catalysis, which are obtained by the weigh-in and the ideal structure formula:

222-Zn: $Zr_6(OH)_8(OH)_8(TCPP\text{-}Zn)_2$; M = 2534.52 g·mol⁻¹

224-Zn: $Zr_6O_{12}(OH)_8(TCPP\text{-}Zn)_{1.5}$; M = 2160.72 g·mol⁻¹

$$n_{222}[\mu\text{mol}] = \frac{m_{\text{Sample}}}{M_{\text{222-Zn}}} = \frac{1.50\ \text{mg}}{2534.52\ \text{g}\cdot\text{mol}^{-1}} = 0.592\ \mu\text{mol}$$

As one structural unit contains one Zr6-oxo node, $n_{\text{222-Zn}} = n_{\text{Node}}$. Exemplarily, the estimated TON after 80 h for **222-Zn** is:

$$TON_{\text{HCOO}^-} = \frac{n_{\text{HCOO}^-,\text{Total}}[\mu\text{mol}]}{n_{\text{Node}}[\mu\text{mol}]} = \frac{3.204\ \mu\text{mol}}{0.592\ \mu\text{mol}} = 5.4$$

Table S4: Headspace measurement data for a typical experiment with **Re-222** under irradiation > 490 nm.

Time (h)	CO (Area%)	CO (Vol%)	$V_{\text{CO,Gas}}$ (mL)	$n_{\text{CO,Gas}}$ (µmol)	p_{CO} (mbar)	$n_{\text{CO,Total}}$ (µmol)	TON
0	0	0	0	0	0	0	0
1.0	0.011	0.013	0.009	0.5	0.1	0.6	4.6
8.0	0.061	0.073	0.052	3.1	0.7	3.1	25.3
24.0	0.154	0.184	0.130	7.8	1.9	7.8	64.1
48.0	0.211	0.252	0.179	10.6	2.6	10.7	87.8
72.0	0.239	0.286	0.202	12.0	2.9	12.1	99.5
80.0	0.243	0.290	0.206	12.2	3.0	12.4	101

Table S5: Incident light flux measurements and calculations. 4 mL 0.15 M potassium ferrioxalate solution. Irradiation time 60.0 s. Addition of 667 µL of 0.1% buffered phenanthroline, UV-Vis measurements after 5 minutes.

Wave-length (nm)	Abs. at 510 nm	c[Fe^{2+}] (M)	n[Fe^{2+}] (mol)	Quantum yield[5]	Light abs.[3]	N/t (mol·s⁻¹)	N photons (s⁻¹)
Dark control	0.057						
450	1.467	$1.27\cdot10^{-4}$	$5.93\cdot10^{-7}$	0.96	1.00	$1.03\cdot10^{-8}$	$6.20\cdot10^{15}$
520	0.254	$1.77\cdot10^{-5}$	$8.29\cdot10^{-8}$	0.71	0.24	$8.11\cdot10^{-9}$	$4.88\cdot10^{15}$

$$c[\text{Fe}^{2+}] = \frac{\text{Abs. at 510 nm} - \text{Abs. dark control}}{\varepsilon \cdot l} = \frac{\text{Abs. at 510 nm} - 0.057}{11100\ \text{M}^{-1}\text{cm}^{-1} \cdot 1\ \text{cm}}$$

$$n[\text{Fe}^{2+}] = c[\text{Fe}^{2+}] \cdot \text{V} = c[\text{Fe}^{2+}] \cdot 0.00467\ \text{L}$$

$$\text{N/t} = \frac{n[\text{Fe}^{2+}]}{\text{Quantum yield} \cdot \text{Irradiation time} \cdot \text{Light abs.}}$$

$$\text{N photons} = \text{N/t} \cdot 6.02215 \cdot 10^{23}$$

$$\text{AQY} = \frac{\text{Amount of product generated per s}}{\text{Number of incident photons per s}} \cdot \text{Number of required electrons}$$

Table S6: AQY calculations. Extracted from kinetic data for catalysis runs with monochromatic LEDs as used in Table S5. Otherwise, standard conditions. Rates averaged over the first 2 h.

Sample	λ (nm)	CO (μmol·g^{-1}·h^{-1})	CO molecules (s^{-1})	AQY_{CO} (%)	Formate (μmol·g^{-1}·h^{-1})	Formate molecules (s^{-1})	AQY_{HCOO^-} (%)
**Re-222**	450	1.1 ± 0.1	(2.8 ± 0.2)·10^{11}	0.01 ± 0.01	12.9 ± 0.6	(3.2 ± 0.1)·10^{12}	0.10 ± 0.01
**Re-222**	520	34.0 ± 0.7	(8.5 ± 0.2)·10^{12}	0.35 ± 0.01	0.4 ± 0.1	(1.1 ± 0.3)·10^{11}	0.01 ± 0.01
**Re-224**	450	0.1 ± 0.1	(2.8 ± 0.2)·10^{11}	0.01 ± 0.01	4.2 ± 0.3	(1.1 ± 0.1)·10^{12}	0.03 ± 0.01
**Re-224**	520	2.9 ± 0.3	(7.3 ± 0.7)·10^{11}	0.03 ± 0.01	0.3 ± 0.1	(8.8 ± 2.9)·10^{10}	0.01 ± 0.01

Table S7: ICP-MS measurement values for Re in the supernatant after photocatalysis under standard conditions with irradiation at > 490 nm, MeCN (4 mL), H_2O (120 μL, 3 vol%) BIH (225 mg, 1 mmol).

Sample	Sample mass (mg)	Supernatant Re (μg)	Supernatant Re (nmol)	Leached Re during catalysis (%)
**Re-222**	0.71	2.86 ± 0.04	15.4 ± 0.2	26.5 ± 0.3
**Re-224**	0.65	1.55 ± 0.02	8.3 ± 0.1	17.0 ± 0.2

5. Bibliography

1 a) J. Rouquerol, P. Llewellyn and F. Rouquerol, in *Characterization of Porous Solids VII - Proceedings of the 7th International Symposium on the Characterization of Porous Solids (COPS-VII), Aix-en-Provence, France, 26-28 May 2005*, Elsevier, 2007, vol. 160, pp. 49–56; b) M. Thommes, K. Kaneko, A. V. Neimark, J. P. Olivier, F. Rodriguez-Reinoso, J. Rouquerol and K. S. Sing, *Pure Appl. Chem.*, 2015, **87**, 1051;

2 S. P. Pitre, C. D. McTiernan, W. Vine, R. DiPucchio, M. Grenier and J. C. Scaiano, *Sci. Rep.*, 2015, **5**, 16397.

3 C. G. Hatchard and C. A. Parker, *Proc. R. Soc. Lond. A*, 1956, **235**, 518.

4 S. Hamai and F. Hirayama, *J. Phys. Chem.*, 1983, **87**, 83.

5 J. W. Greenberg, V. Malhotra and J. F. Ennever, *Photochem. Photobiol.*, 1987, **46**, 453.

6 a) Y.-P. Chen, Y. Liu, D. Liu, M. Bosch and H.-C. Zhou, *J. Am. Chem. Soc.*, 2015, **137**, 2919; b) A. A. Yakovenko, J. H. Reibenspies, N. Bhuvanesh and H.-C. Zhou, *J. Appl. Crystallogr.*, 2013, **46**, 346; c) A. A. Yakovenko, Z. Wei, M. Wriedt, J.-R. Li, G. J. Halder and H.-C. Zhou, *Cryst. Growth Des.*, 2014, **14**, 5397;

7 E. Portenkirchner, J. Gasiorowski, K. Oppelt, S. Schlager, C. Schwarzinger, H. Neugebauer, G. Knör and N. S. Sariciftci, *ChemCatChem*, 2013, **5**, 1790.

8 Z. K. Lopez-Castillo, Aki, Sudhir N. V. K., M. A. Stadtherr and J. F. Brennecke, *Ind. Eng. Chem. Res.*, 2006, **45**, 5351.

9 E. Rousset, D. Chartrand, I. Ciofini, V. Marvaud and G. S. Hanan, *Chem. Commun.*, 2015, **51**, 9261.

10 P. Wolf, S. L. Heath and J. A. Thomas, *Inorganica Chim. Acta*, 2003, **355**, 280.

11 B. W. Pfennig, P. Chen and T. J. Meyer, *Inorg. Chem.*, 1996, **35**, 2898.

12 D. Feng, Z.-Y. Gu, J.-R. Li, H.-L. Jiang, Z. Wei and H.-C. Zhou, *Angew. Chem. Int. Ed.*, 2012, **51**, 10307.

13 X. Gong, Y. Shu, Z. Jiang, L. Lu, X. Xu, C. Wang and H. Deng, *Angew. Chem. Int. Ed.*, 2020, **59**, 5326.

14 K. Epp, A. L. Semrau, M. Cokoja and R. A. Fischer, *ChemCatChem*, 2018, **10**, 3506.

15 a) J. Jin, *Reac. Kinet. Mech. Cat.*, 2020, **131**, 397; b) J. Liang, Y.-Q. Xie, Q. Wu, X.-Y. Wang, T.-T. Liu, H.-F. Li, Y.-B. Huang and R. Cao, *Inorg. Chem.*, 2018, **57**, 2584;

16 G. Kickelbick and U. Schubert, *Chem. Ber.*, 1997, **130**, 473.

17 A. L. Semrau and R. A. Fischer, *Chem. Eur. J.*, 2021, **27**, 8509.

18 G. Lucazeau, L. Guemas and A. Novak, *Inorganica Chim. Acta*, 1976, **20**, 11.

19 a) Y. Pellegrin and F. Odobel, *C. R. Chimie*, 2017, **20**, 283; b) J. Jin, *New J. Chem.*, 2020, **44**, 15362;

6. Atomic coordinates for DFT calculated molecules

$ReBr(CO)_3(qtpy)$ (**1**) (xyz coordinates in Å)

C	-0.65786	2.67108	0.34014
C	0.46361	3.49085	0.25083
C	1.74115	2.91197	0.15895
C	1.82160	1.51156	0.16530
C	0.66005	0.73959	0.25515
N	-0.56912	1.32078	0.34236
C	2.96383	3.74960	0.05602
C	2.97707	4.92258	-0.71980
C	4.15382	5.68066	-0.79015
N	5.29196	5.34440	-0.14299
C	5.27596	4.21785	0.60372
C	4.14713	3.39747	0.73035
C	0.65998	-0.73960	0.25519
C	1.82144	-1.51169	0.16525
C	1.74087	-2.91209	0.15905
C	0.46328	-3.49082	0.25118
C	-0.65811	-2.67094	0.34051
N	-0.56925	-1.32065	0.34256
C	2.96345	-3.74988	0.05602
C	2.97613	-4.92347	-0.71889
C	4.15276	-5.68172	-0.78934
N	5.29133	-5.34506	-0.14313
C	5.27588	-4.21792	0.60269
C	4.14720	-3.39734	0.72934
Re	-2.28484	0.00014	0.31373
Br	-1.81345	-0.00007	-2.33155
C	-2.47921	0.00027	2.20012
O	-2.59253	0.00053	3.37930
C	-3.63273	1.34692	0.11923
O	-4.43293	2.20297	-0.00762
C	-3.63283	-1.34655	0.11946
O	-4.43312	-2.20257	-0.00706
H	-1.65055	3.09258	0.40842
H	0.33635	4.56596	0.27088
H	2.78798	1.03455	0.06556
H	2.10380	5.23254	-1.28260
H	4.19504	6.58559	-1.38556
H	6.19939	3.97537	1.11741
H	4.19006	2.52101	1.36767
H	2.78781	-1.03473	0.06525
H	0.33587	-4.56591	0.27146
H	-1.65082	-3.09235	0.40896
H	2.10255	-5.23379	-1.28098
H	4.19353	-6.58714	-1.38405
H	6.19964	-3.97511	1.11564
H	4.19064	-2.52040	1.36598

Re-222 pore fragment

O -4.97916 -9.57989 1.79730
O -3.89343 -8.97551 3.64432
N -4.23081 -1.95777 -1.12986
N -4.52244 -0.85736 1.55310
C -4.50492 -8.72602 2.58770
C -4.61880 -7.29354 2.25300
C -3.70665 -6.39837 2.78732
H -3.06009 -6.69154 3.42020
C -3.74236 -5.06947 2.38789
H -3.12368 -4.45460 2.75629
C -4.67412 -4.63265 1.45681
C -5.60063 -5.53468 0.94784
C -5.55329 -6.87560 1.33537
H -6.16653 -7.49710 0.96344
C -4.61425 -3.26018 0.94295
C -4.66267 -2.18899 1.87054
C -4.91069 -2.29953 3.24204
H -5.05414 -3.10749 3.71956
C -4.31940 -3.12378 -0.41377
C -3.95806 -4.19307 -1.29277
H -3.89958 -5.11177 -1.05597
C -3.91779 -2.27375 -2.42694
C -4.67951 -0.15501 2.72601
H -6.26629 -5.24207 0.33471
C -4.91221 -1.06195 3.76429
C -3.72010 -3.68940 -2.48564
C -3.73012 -1.38885 -3.48900
C -4.56909 1.25240 2.85734
H -5.04629 -0.84141 4.67794
H -3.46081 -4.18308 -3.25542
C -3.42795 -1.99502 -4.78998
C -3.61970 0.01855 -3.35766
C -4.87125 1.85857 4.15832
C -4.38141 2.13730 1.79528
C -2.19169 -1.78734 -5.38530
C -4.33588 -2.85772 -5.39210
N -3.77677 0.72091 -2.18476
C -3.38699 0.92551 -4.39594
C -6.10752 1.65090 4.75365
C -3.96333 2.72127 4.76044
C -4.57911 3.55295 1.85400
N -4.06839 1.82132 0.49821
C -1.84369 -2.45525 -6.55132
H -1.57780 -1.18262 -4.99286
C -3.97924 -3.54399 -6.55500
H -5.19934 -2.98378 -5.01368
C -3.63653 2.05253 -2.50219
H -3.25291 0.70496 -5.30960
C -3.38851 2.16309 -3.87369

H -6.72140 1.04618 4.36121
C -6.45550 2.31880 5.91966
H -3.09986 2.84733 4.38202
C -4.31996 3.40754 5.92334
H -4.83839 4.04663 2.62376
C -4.34114 4.05663 0.66113
C -3.97981 2.98733 -0.21788
C -2.74744 -3.33266 -7.12771
H -0.99153 -2.31325 -6.94905
H -4.58828 -4.15661 -6.94797
C -3.68496 3.12374 -1.57461
H -3.24507 2.97103 -4.35121
H -7.30766 2.17681 6.31740
C -5.55176 3.19621 6.49605
H -3.71092 4.02016 6.31632
H -4.39962 4.97532 0.42431
C -2.33326 -4.12952 -8.29836
C -3.62508 4.49620 -2.08847
C -5.96595 3.99306 7.66671
O -1.38113 -3.65801 -8.94932
O -2.91731 -5.21581 -8.53830
C -4.55685 4.93302 -3.01954
C -2.69857 5.39823 -1.57950
O -5.38189 5.07937 7.90664
O -6.91808 3.52156 8.31766
H -5.17553 4.31815 -3.38794
C -4.59254 6.26192 -3.41897
H -2.03291 5.10562 -0.96637
C -2.74591 6.73915 -1.96703
C -3.68040 7.15710 -2.88466
H -5.23910 6.55509 -4.05186
H -2.13267 7.36065 -1.59509
C -3.79429 8.58956 -3.21935
O -3.32004 9.44344 -2.42896
O -4.40577 8.83906 -4.27598
Zn -3.90764 -0.09350 -0.27834
H -3.43223 10.37088 -2.71164
H -1.07778 -4.19686 -9.70443
H -3.78547 -9.92222 3.85588
H -5.70817 5.55492 8.69404
Re 7.41103 -1.28576 1.25378
Br 7.22032 -1.70068 -1.39356
O 10.51495 -1.16883 1.07343
O 7.50790 -4.35060 1.77815
O 7.45903 -0.65824 4.28132
N 5.52327 7.24900 -1.86683
N 7.04655 0.77011 0.68090
N -1.72260 -0.38184 0.03342
N 5.25741 -1.12329 1.11191
C 9.32391 -1.23722 1.15431
C 7.49647 -3.17103 1.58302

C 7.44463 -0.90355 3.11178
C 4.97667 5.38014 -0.42676
C 4.71870 6.64936 -0.96152
C 6.62714 6.57671 -2.26264
C 6.96649 5.30528 -1.78045
C 6.12638 4.68258 -0.83985
C 8.02718 1.67921 0.47336
C 7.76727 2.95730 -0.01278
C 6.44483 3.33377 -0.30482
C 5.43574 2.38553 -0.08107
C 5.75732 1.11666 0.40859
C 0.37125 0.52134 0.79962
C -1.00305 0.62691 0.56991
C -1.09508 -1.53243 -0.29270
C 0.27596 -1.71626 -0.09276
C 1.03576 -0.67241 0.46472
C 4.75458 0.05834 0.65686
C 3.38335 0.22761 0.44469
C 2.49538 -0.82878 0.69252
C 3.03444 -2.03957 1.15951
C 4.40801 -2.14806 1.35646
H 4.30556 4.96569 0.31760
H 3.84413 7.21278 -0.65628
H 7.25228 7.07728 -2.99313
H 7.85295 4.80754 -2.15700
H 9.03628 1.36488 0.69827
H 8.58818 3.65093 -0.14470
H 4.41073 2.63342 -0.32471
H 0.90033 1.34861 1.25854
H -1.56030 1.52294 0.81834
H -1.71012 -2.31209 -0.72587
H 0.73847 -2.64882 -0.39363
H 3.00189 1.16311 0.05638
H 2.39825 -2.88594 1.38631
H 4.84781 -3.06837 1.71298

Re-224 pore fragment

O -12.37719 -0.26654 -7.34011
O -2.34146 -5.12450 8.53019
O -12.43652 4.26187 5.05176
O -2.28214 -9.65291 -3.86168
O 12.87375 -0.12719 -7.27015
O 2.80446 -5.09610 8.54444
O 12.81442 4.40122 5.12172
O 2.86379 -9.62451 -3.84742
O 10.84589 5.72834 5.17200
O 1.62026 -7.96506 -5.00447
O 1.55634 -3.08547 8.34839
O 10.90981 0.84875 -8.18086
O -10.48306 5.61063 5.11291

O -1.05061 -7.97980 -5.01187
O -1.11454 -3.10021 8.34099
O -10.41913 0.73105 -8.23996
C -11.09826 0.09752 -7.28682
C -2.02791 -3.97761 7.93238
C -11.15606 4.50974 4.78712
C -1.97010 -8.38984 -4.14156
C 11.59060 0.22272 -7.22396
C 2.48158 -3.95273 7.94487
C 11.53280 4.63495 4.84998
C 2.53938 -8.36495 -4.12906
C -10.37253 -0.22854 -6.06911
C -2.75364 -3.65156 6.71467
C -10.42108 3.47762 4.07272
C -2.70509 -7.35772 -3.42716
C 10.86177 -0.11136 -6.01028
C 3.21041 -3.61864 6.73119
C 10.81322 3.59481 4.13155
C 3.25896 -7.32481 -3.41064
C -11.00139 -0.93670 -5.04504
C -3.76169 -4.49647 6.25031
C -11.04345 2.27411 3.74125
C -3.71962 -7.70728 -2.53599
C 11.49272 -0.81257 -4.98272
C 4.23027 -4.45237 6.27245
C 11.45066 2.39824 3.80357
C 4.27233 -7.66318 -2.51384
C 9.48095 3.78700 3.76580
C 2.94041 -5.98206 -3.61371
C 2.89431 -2.46232 6.01798
C 9.52706 0.26726 -5.86589
C -9.08900 3.68452 3.71435
C -2.40025 -6.01154 -3.62850
C -2.44636 -2.49180 6.00319
C -9.04289 0.16477 -5.91734
H -11.90477 -1.21281 -5.14914
H -3.97847 -5.28317 6.73743
H -11.94853 2.12739 3.99121
H -3.93471 -8.62336 -2.40292
H 12.39967 -1.07868 -5.08180
H 4.45303 -5.23664 6.76079
H 12.35591 2.26151 4.05855
H 4.49678 -8.57683 -2.37956
H 9.04663 4.60445 3.98101
H 2.24504 -5.74913 -4.21817
H 2.19457 -1.89669 6.32394
H 9.09709 0.75201 -6.56110
H -8.66493 4.50671 3.93194
H -1.70416 -5.77093 -4.22911
H -1.75463 -1.91849 6.31300
H -8.61446 0.65426 -6.61017

C -10.31101 -1.23632 -3.87975
C -4.44447 -4.18965 5.08242
C -10.34418 1.29589 3.04957
C -4.41130 -6.72186 -1.84690
C 10.79926 -1.11983 -3.82126
C 4.91610 -4.13800 5.10836
C 10.76608 1.41238 3.10806
C 4.94928 -6.67021 -1.82096
C 8.79310 2.78903 3.09108
C 3.63589 -4.99121 -2.93635
C 3.59853 -2.13951 4.86727
C 8.83046 -0.06267 -4.71254
C -8.38645 2.69423 3.04349
C -3.11038 -5.02844 -2.95505
C -3.14774 -2.17673 4.84858
C -8.34910 -0.15748 -4.76014
H -10.74883 -1.70373 -3.17865
H -5.11815 -4.77684 4.76138
H -10.77750 0.48522 2.81136
H -5.08948 -6.96579 -1.22862
H 11.23832 -1.58238 -3.11773
H 5.59799 -4.71770 4.79107
H 11.20964 0.60657 2.87227
H 5.62666 -6.90665 -1.19893
H 7.88131 2.91802 2.85938
H 3.42674 -4.07794 -3.09090
H 3.39091 -1.34242 4.39479
H 7.91715 0.18249 -4.62631
H -7.47487 2.83327 2.81684
H -2.91046 -4.11291 -3.10846
H -2.94630 -1.37739 4.37724
H -7.43904 0.09775 -4.66885
C -8.98169 -0.85390 -3.73461
C -4.14473 -3.02641 4.38026
C -9.01259 1.49863 2.70336
C -4.11393 -5.37907 -2.05768
C 9.46418 -0.75142 -3.68381
C 4.60738 -2.97802 4.40451
C 9.43361 1.60102 2.75428
C 4.63821 -5.33067 -2.03351
C -8.29789 -1.16152 -2.58701
C -4.82979 -2.71996 3.23249
C -8.32052 0.52587 2.03050
C -4.80774 -4.40704 -1.38423
C 8.77660 -1.06611 -2.54016
C 5.29385 -2.66262 3.26064
C 8.75454 0.62094 2.07716
C 5.31585 -4.35005 -1.35672
C -7.62319 -2.48977 -2.45488
C -6.13144 -1.98468 3.31574
C -7.64642 -0.57381 2.79073

C -6.10547 -3.90330 -1.93100
C 8.68205 0.66127 0.58128
C 4.75693 -3.80377 -0.08195
C 4.74962 -3.06987 1.92951
C 8.69182 -0.07109 -1.42318
C 7.49177 -1.93849 -2.66625
C 6.05594 -1.30502 3.20680
C 7.46598 0.03244 2.72455
C 6.08296 -3.27499 -2.18390
C -8.86496 0.05084 0.64587
C -4.77977 -4.42318 0.17634
C -4.78982 -3.73897 2.05117
C -8.85601 -0.63355 -1.22731
H -9.51661 0.94576 -8.10832
H -12.82299 3.45273 4.77992
H 13.21131 3.59640 4.85205
H 10.00427 1.05349 -8.05423
H -0.90265 -7.06350 -5.14023
H 1.46290 -7.05044 -5.13367
H -3.00243 -5.67842 8.16376
H 3.47352 -5.64268 8.18171
C -7.50080 -3.36931 -3.60356
C -6.53781 -1.37189 4.56916
C -7.50654 -0.47199 4.23390
C -6.53119 -4.27090 -3.26947
C 9.54826 1.56257 -0.15239
C 3.70267 -4.53902 0.59623
C 3.69832 -4.07231 1.87716
C 9.55482 1.09405 -1.43600
H -8.07913 -3.29576 -4.51138
H -6.12934 -1.60554 5.53993
H -8.06939 0.19258 4.87063
H -6.14281 -5.09808 -3.84316
H 10.07262 2.40652 0.26810
H 3.09040 -5.30644 0.14872
H 3.08082 -4.37137 2.70985
H 10.08891 1.46803 -2.29569
C 7.36254 -2.71638 -3.86920
C 6.32740 -0.65813 4.46185
C 7.28658 0.24820 4.13484
C 6.40483 -3.62260 -3.54232
C -9.83321 0.89120 -0.00432
C -3.80427 -5.25409 0.82238
C -3.81080 -4.78723 2.10088
C -9.82692 0.42491 -1.28174
H 7.94710 -2.60318 -4.76621
H 5.88392 -0.89952 5.41269
H 7.83488 0.94120 4.74975
H 5.99913 -4.44857 -4.10095
H -10.38012 1.70231 0.44452
H -3.21627 -6.02898 0.36168

H -3.22754 -5.07871 2.95699
H -10.37012 0.75036 -2.15216
N -8.28933 -0.89888 -0.06656
N -6.84860 -1.52844 2.26675
N -5.40141 -3.51472 0.90381
N -6.80846 -2.85685 -1.44261
N 6.69526 -2.23306 -1.65405
N 8.14168 -0.28827 -0.21133
N 6.70876 -0.88011 2.14283
N 5.32964 -2.88005 0.72178
Zn 6.50614 -1.36745 0.17357
Zn -6.55857 -1.93308 0.31914
C 2.27845 6.15702 -0.64167
C 3.14725 5.07573 -0.51224
C 2.62165 3.78280 -0.34891
C 1.22850 3.64721 -0.29904
C 0.40801 4.76823 -0.43709
N 0.93271 6.01147 -0.61944
C 3.49270 2.58284 -0.26737
C 4.64622 2.47375 -1.06360
C 5.42123 1.31400 -0.98564
N 5.09774 0.28589 -0.17234
C 3.99558 0.38148 0.60200
C 3.17277 1.50906 0.58451
C -1.06657 4.69517 -0.43306
C -1.77225 3.50321 -0.25804
C -3.17138 3.49924 -0.31741
C -3.82355 4.72579 -0.52599
C -3.06599 5.88621 -0.67216
N -1.71263 5.87629 -0.63773
C -3.91530 2.21969 -0.19958
C -4.96584 1.91646 -1.08250
C -5.60938 0.68202 -0.97568
N -5.26376 -0.23136 -0.04128
C -4.26481 0.05511 0.82221
C -3.56527 1.26259 0.77053
Re -0.46945 7.58944 -1.09885
Br -0.38827 6.42462 -3.52274
C -0.52085 8.28248 0.66740
O -0.55227 8.70573 1.77164
C 0.81871 8.90174 -1.63309
O 1.64090 9.67743 -1.96370
C -1.87369 8.76527 -1.66154
O -2.76316 9.45397 -2.00968
H 2.65613 7.15995 -0.78117
H 4.21570 5.25084 -0.53312
H 0.79212 2.66201 -0.20135
H 4.93025 3.26082 -1.75175
H 6.32264 1.19626 -1.57591
H 3.78884 -0.46332 1.25008
H 2.31654 1.55372 1.24732

H -1.24571 2.56807 -0.11973
H -4.90407 4.78852 -0.56422
H -3.54011 6.84382 -0.83317
H -5.26516 2.61322 -1.85626
H -6.41834 0.40597 -1.64282
H -4.04548 -0.69945 1.57016
H -2.78436 1.46070 1.49551

7. SUPERFLIP Input File

Exemplary input file in .inflip format for **Re-222**.

```
title Re-PCN-222-Zn
# crystallographic data
#---------------------
cell 39.2630 39.2630 16.5666 90.000 90.000 120.000
# spacegroup P6/mmm
spacegroup P6/mmm
centro yes
centers
  0.000000  0.000000  0.000000
endcenters
symmetry
     x1     x2     x3
    -x2  x1-x2     x3
 -x1+x2    -x1     x3
    -x1    -x2     x3
     x2 -x1+x2     x3
  x1-x2     x1     x3
     x2     x1    -x3
  x1-x2    -x2    -x3
    -x1 -x1+x2    -x3
    -x2    -x1    -x3
 -x1+x2     x2    -x3
     x1  x1-x2    -x3
    -x1    -x2    -x3
     x2 -x1+x2    -x3
  x1-x2     x1    -x3
     x1     x2    -x3
    -x2  x1-x2    -x3
 -x1+x2    -x1    -x3
    -x2    -x1     x3
 -x1+x2     x2     x3
     x1  x1-x2     x3
     x2     x1     x3
  x1-x2    -x2     x3
    -x1 -x1+x2     x3
endsymmetry
# Grid definition for density maps
#-------------------------------
dimension 3
```

```
voxel AUTO
# control parameters for Superflip run
#-----------------------------
maxcycles 10000
repeatmode 1
bestdensities 1
outputfile Re-PCN-222-Zn.xplor
expandedlog yes

# Keywords for charge flipping
#-----------------------------
perform fourier
delta AUTO
weakratio 0.0
Biso 0.0
randomseed AUTO
searchsymmetry average
# input data
#-----------
dataformat intensity phase
fbegin
# h k l F^2obs phase
 1 0 0 7162.23 0.0
 2 0 0 1826.73 0.0
 3 -1 0 58.62 0.0
 2 -1 1 181.50 0.0
 2 0 1 436.30 0.0
 3 0 0 218.77 0.0
 3 -1 1 66.75 0.0
 4 0 0 238.42 0.0
endf
```
